# Topical application of the antimuscarinic pirenzepine increased lower limb nerve fibre density in a phase 2a study in type 2 patients with diabetes with peripheral neuropathy

**DOI:** 10.1016/j.ebiom.2025.106055

**Published:** 2025-12-05

**Authors:** Ajith Sivadasan, Paul Fernyhough, Nigel A. Calcutt, Katie E. Frizzi, Kimberly Gardner, Angela Hansen, Ari Breiner, Douglas W. Zochodne, Natalia McInnes, Zubin Punthakee, Sylvie Gosselin, Bruce A. Perkins, Vera Bril

**Affiliations:** a Ellen & Martin Prosserman Centre for Neuromuscular Diseases, Toronto General Hospital, University Health Network, University of Toronto, Toronto, Ontario, Canada; b Division of Neurodegenerative Disorders, St. Boniface Hospital Albrechtsen Research Centre, Winnipeg, Manitoba, Canada; c Canada and Department of Pharmacology & Therapeutics, University of Manitoba, Manitoba, Canada; d Department of Pathology, University of California San Diego, La Jolla, CA, USA; e WinSanTor Inc, 7220 Trade St, Ste. 370, San Diego, CA 92121, USA; f Division of Neurology, Department of Medicine, The Ottawa Hospital and Ottawa Hospital Research Institute, Ottawa, Ontario, Canada; g Division of Neurology, Department of Medicine, Alberta Diabetes Institute, Neuroscience and Mental Health Institute, University of Alberta, Edmonton, Alberta, Canada; h McMaster University, 1200 Main St W., Borris clinic office 4Z35, Hamilton, ON L8S 2A, Canada; i Service de Neurologie, Centre Intégré Universitaire de Santé et de Services Sociaux de l’Estrie – Centre Hospitalier Universitaire de Sherbrooke 3001, 12e Avenue Nord, Sherbrooke (Qc) J1H 5N4, Canada; j Leadership Sinai Centre for Diabetes, Mount Sinai Hospital, Sinai Health L5-210, 60 Murray Street, Mail Box 16, Toronto, Ontario, M5T 3L9, Canada

**Keywords:** Diabetic neuropathy, Treatment, Pirenzepine, Clinical trial

## Abstract

**Background:**

There is no effective definitive treatment for diabetic peripheral neuropathy (DPN). Strategies that target nerve pathology, limit nerve degeneration, and promote axon regeneration can potentially be beneficial. Topical pirenzepine promotes nerve fibre protection and repair in rodent models of type 1 and type 2 diabetes. We assessed the efficacy and safety of topical pirenzepine for the treatment of DPN.

**Methods:**

Preclinical studies were performed to determine efficacy of a topical formulation of 4% pirenzepine against indices of neuropathy in adult female Sprague-Dawley rats with streptozotocin (STZ)-induced diabetes. We then did a proof-of-concept randomised Phase 2a, double-blind, placebo-controlled, clinical trial conducted across 5 university centres in Canada. Participants (aged 18–75 years) with definite type-2 DPN, as defined by the Toronto Consensus Guidelines, who were on stable anti-diabetic and analgesic therapy prior to screening and during the study were enrolled. Participants were randomised to 1 of 4 treatment groups in a 1:1:4:4 ratio, where placebo (2 mL), placebo (4 mL); pirenzepine (2 mL of 4% formulation); and pirenzepine (4 mL of 4% formulation) were self-administered topically by the participants for a 24 weeks period. Safety and tolerability of 2 dose levels of 4% pirenzepine for 24 weeks was the primary objective. The primary efficacy endpoints were the change at 24 weeks from baseline in the intraepidermal nerve fibre density (IENFD) collected from the treated skin (ankle) and the impact on the Norfolk-quality of life-diabetic neuropathy (QOL-DN). Punch biopsies (3 mm) were collected and shipped to a centralised facility for processing and reading. The trial was approved by Health Canada (226063) and registered with ClinicalTrials.govNCT04005287.

**Findings:**

In rats, topically administered 4% pirenzepine (100 μl) to the hind paws applied 5 days per week for 6 weeks of diabetes prevented large fibre conduction slowing, touch-evoked allodynia, reduced mean axonal diameter, and small fibre mediated heat hypoalgesia and cold hyperalgesia. Two doses of topical pirenzepine or placebo were administered to 58 DPN individuals for 24 weeks, between October 15, 2019 and December 15, 2021. The least squares mean difference in change from baseline to week 24 in the IENFD at the ankle was 2.32 (p = 0.006) in the pirenzepine 4 mL group; 1.50 (p = 0.048) in the pirenzepine 2 mL group and −0.71 (p = 0.39) in placebo patients on the modified-intent-to-treat analysis (mITT). The change in IENFD at the ankle was significant in the combined pirenzepine groups compared to placebo (p = 0.012). No differences were observed in other parameters in the mITT population. There was a 10.4-point improvement in the Norfolk QOL-DN score in the combined treatment groups over placebo (p < 0.001) in the per protocol (PP) analysis set, but no difference was observed in the modified intention-to-treat analysis, suggesting the need for cautious interpretation of the PP result due to potential bias. Systemic adverse events with possible/probable/definite relation to study drug were similar between the treatment groups. Administration site reactions were the most common reported treatment emergent adverse events among the pirenzepine treatment groups (pirenzepine 4 mL 41.7%, pirenzepine 2 mL 22.6% and placebo 8.3%). There were no deaths.

**Interpretation:**

The efficacy of topical pirenzepine in preclinical studies was translated to a clinical study. The overall systemic safety profile was similar between patient groups. However, local topical reactions were more prevalent in the active dose groups than in the placebo group. Topical pirenzepine administered once daily for 24 weeks in patients with DPN resulted in a statistically significant growth of nerve fibres as measured by IENFD at the ankle. This study in humans with DPN demonstrates that selective targeting of the muscarinic acetylcholine type 1 receptor promotes regeneration of distal axons. Future phase 2b, and phase 3 studies are needed to substantiate these observations.

**Funding:**

The study was funded by the 10.13039/501100000024CIHR, 10.13039/100008794Research Manitoba and WinSanTor.

**Research in context**

**Evidence before this study**

We searched PubMed up to October 2025, for relevant clinical studies in diabetic peripheral neuropathy (DPN), with no language restrictions. Key search terms included “diabetic neuropathy” and the article type selected was “randomised controlled trial”. One publication in JPNS 2025, with dapaglifozin showed small fibre regeneration in DPN. Currently, there are no approved disease-modifying treatments for DPN. The available therapies are symptomatic, for relieving pain. There remains a need for disease-modifying treatments that limit axonal degeneration and promote axonal regeneration and nerve recovery in DPN. Drugs exerting antimuscarinic activity, including the selective orthosteric antagonist pirenzepine, have been identified as stimulators of neurite outgrowth in cultured adult rat sensory neurons. Indices of peripheral neuropathy such as depletion of sensory nerve terminals (in foot skin or cornea), thermal hypoalgesia, tactile allodynia and sciatic nerve conduction slowing were either prevented or reversed in rodent models of type 1 and type 2 diabetes, critical illness polyneuropathy or HIV-neuropathy by systemic or topical application of antimuscarinic agents such as pirenzepine.

**Added value of this study**

Building on preclinical evidence, this phase 2 randomised trial examines the use of the muscarinic antagonist, pirenzepine, in DPN. Topical pirenzepine administered once daily for 24 weeks in patients with DPN had a significant positive effect on the intraepidermal nerve fibre density (IENFD) at the calf. There were trends showing improvement noted in the clinical outcome measures such as Norfolk quality of life diabetic neuropathy (QOL-DN) score and modified Toronto Neuropathy score (mTCNS). The safety profile was similar in all the treatment groups.

**Implications of all the available evidence**

This study in humans with DPN demonstrates that targeting of a specific signal transduction pathway, namely muscarinic receptor antagonism, can suppress the dying-back of distal axons. The positive effect of topical pirenzepine on IENFD at the ankle was seen over a relatively short period of time, and this initial observation is encouraging and has implications for other proof-of-concept studies in patients with DPN. This study was not specifically powered to detect functional recovery, and hence larger studies over a longer period of treatment might be required to study the impact of our observations on the eventual clinical outcome in DPN.

## Introduction

Diabetic peripheral neuropathy (DPN) is a major, untreated morbidity associated with high health care costs.^1^ There are approximately 537 million people with diabetes worldwide,^2^ over half of whom will develop some form of nerve dysfunction in their lifetime.^3^ Much of the recent focus has centred on the development of therapies to address painful symptoms which occurs in nearly 33–50% of patients with DPN.^3^, ^4^, ^5^ However, of individuals diagnosed with DPN, over two-thirds present with neuropathic symptoms other than pain.^4^ These often-ignored painless diabetic neuropathies can lead to ulcerations, foot deformities, Charcot joints and recurrent infection leading to gangrene and amputation. While optimal glycaemic control is encouraged, evidence that it can slow progression of DPN in type 2 diabetes mellitus is limited.^6^ The immense physical, psychological, and economic costs of DPN underscore the unmet need for effective therapies that address the underlying pathology associated with nerve degeneration.^7^

To identify novel compounds with nerve growth promoting properties, a drug screen using previously FDA-approved compounds was performed in cultured adult rat sensory neurons, a model system for studying mechanisms of collateral sprouting and axon regeneration.^8^^,^^9^ Several drugs exerting antimuscarinic activity, including the muscarinic acetylcholine type 1 receptor (M_1_R) selective orthosteric antagonist pirenzepine, were identified as stimulators of neurite outgrowth in cultured adult sensory neurons.^8^ The efficacy of antimuscarinic agents in vitro was mediated via antagonism of M_1_R and disruption of an endogenous autocrine/paracrine acetylcholine-dependent constraint mechanism that inhibited AMP- activated protein kinase (AMPK), a key enhancer of neuronal bioenergetics,^8^^,^^10^ leading to suppression of mitochondrial function and inhibition of axonal outgrowth and regeneration of sensory neurons.^8^^,^^11^ Pharmacological blockade of M_1_R by pirenzepine or other anti− muscarinic agents in vivo alleviated mitochondrial dysfunction in mouse models of type 1 and type 2 diabetes. Efficacy was accompanied by prevention and/or reversal of multiple indices of peripheral neuropathy that are common to diabetic rodents and humans including depletion of sensory nerve terminals in foot skin or cornea, thermal hypoalgesia, tactile allodynia and sciatic nerve large fibre conduction slowing.^8^^,^^11^, ^12^, ^13^, ^14^

Pirenzepine is a well characterised M_1_R antagonist with limited systemic distribution after oral delivery and minimal penetration of the blood brain barrier.^15^^,^^16^ It has been safely used for other indications such as peptic ulcers in adults and myopia in children.^17^, ^18^, ^19^ Topical delivery of other antimuscarinics is currently used to treat overactive bladder.^20^^,^^21^ Topical application to the sites maximally affected by diabetic neuropathy (below mid-calves including ankles and feet) can maximise local drug concentration without significantly increasing systemic absorption, thus potentially enhancing local efficacy and minimising systemic side effects. In mice, topical pirenzepine showed a pharmacokinetic profile that matches concentrations that promote increased neurite outgrowth from rat or mouse adult sensory neurons in vitro.^8^^,^^12^ Preclinical studies using concentrations of pirenzepine between 0.1% and 10% in hydrogel have shown positive effects in preventing and reversing sensory neuropathy in a mouse model of type 1 diabetes when delivered topically to the hind paw.^12^ We therefore first determined whether efficacy was retained when pirenzepine (4%) was delivered in a vehicle developed for clinical use. Based on this preclinical data, we then translated those findings into the clinic and assess the safety of two topical dose levels of 4% pirenzepine (2 mL, 73 mg pirenzepine free base monohydrate; and 4 mL, 146 mg pirenzepine free base monohydrate, respectively), in patients with DPN in a phase 2 randomised clinical trial.

## Methods

### Preclinical studies

The study was performed using 25 age-matched adult female Sprague-Dawley rats (Envigo, now Inotiv, USA: Cat. # 002) under a protocol approved by the local IACUC. Group size was determined by prior experience. Rats were maintained 2 per cage as per vivarium requirements under a 12 h light:dark cycle with unrestricted access to water and food (Harlan 5001). Insulin-deficient diabetes was induced in 17 randomly selected rats by a single injection of steptozotocin (STZ: 50 mg/kg i.p.) following an overnight fast. Non-fasted blood glucose concentration was measured 5 days after STZ injection and at study end using tail vein blood and a glucose metre (OneTouch Ultra, LifeScan, PA, USA). Only rats with blood glucose values > 15 mmol/l at both points were considered diabetic and included in statistical analyses (N = 16 of 17 at study end). After confirmation of hyperglycemia, diabetic rats were treated (5 days/week for 6 weeks) by application of 100 μl of pirenzepine topical solution (4% w/v% in a vehicle comprising inactive emulsifiers, solvents, solubilizers, penetration enhancers, solvents and thickening agents, subsequently designated as WST-057: N = 9) or vehicle (N = 8) alone to one hind paw. Once applied, the cream was covered to prevent ingestion and left in place for 30 min before wiping away the residuum. Non-diabetic (control) rats (N = 8) were treated with vehicle. Rats were observed daily and weighed weekly during the study. Any rats losing >20% of starting body weight were referred for inspection by a veterinarian and treated daily with insulin (0.5–2IU/day) to alleviate weight loss without impacting hyperglycemia.

At study end, indices of nerve function and structure that model human diabetic neuropathy were measured in coded animals/tissue by observers unaware of the coding key. Measurements were made in a randomly selected sequence of cages. All experimental procedures have been described in technical detail elsewhere.^22^ Briefly, hind paw sensitivity to escalating heat was measured in unrestrained rats using a Hargreaves apparatus (UARD, CA, USA) with floor temperature increasing at 1 °C/s from 30 °C until paw removal (response latency in seconds), with a cut-off at 20 s. Hind paw sensitivity to pressure applied to the plantar surface was measured in unrestrained rats using von Frey filaments. Hind paw sensitivity to cold was measured using a blunt probe cooled to 10 °C by a Peltier system that was placed against the plantar surface until paw withdrawal (positive response) or until 5 s had elapsed (negative response) and the positive response frequency of 10 consecutive applications was determined. Large fibre motor and sensory nerve conduction velocity was measured in the sciatic nerve of isoflurane anaesthetised rats following stimulation (5–10 V, 0.05 mS) at the sciatic notch and Achilles tendon via needle electrodes and recording of the evoked M and H waves in an electromyogram collected from the interosseus muscles of the ipsilateral foot. At autopsy, the distal tibial nerve was removed to 2.5% glutaraldehyde and processed to resin blocks before cutting 1 μm sections that were osmicated, stained with p-phenylenediamine and viewed under a light microscope. Axonal and fibre diameter of all viable myelinated fibres in the tibial nerve was quantified using custom-designed in-house image analysis software (MarvoQuant v 2.2) and g-ratio (ratio of axonal diameter to myelinated fibre diameter) calculated.

### Clinical study design and participants

We performed a randomised phase 2, double-blind, placebo-controlled, clinical trial in 5.

University centres in Canada (University Health Network, University of Toronto; The Ottawa Hospital and Ottawa Hospital Research Institute, University of Ottawa; McMaster University; the Alberta Diabetes Institute, University of Alberta; Centre intégré universitaire de santé et de services, Sociaux de l’Estrie – Centre hospitalier universitaire de Sherbrooke). Eligible patients were men or women with type 2 diabetes mellitus, aged between 18 and 75 years with definite DPN, based on the Toronto Consensus guidelines, of at least 12 months duration in the lower extremities.^23^ All study participants were required to have optimal glycaemic control with stable anti-diabetic treatment (stable for 3 months and not expected to change during the course of the study) and stable analgesic/non-pharmacological therapies (at least 4 weeks prior to screening and during the study). An IENFD at the ankle of 1–10/mm at baseline was required for enrolment. Subjects with uncontrolled glycaemia and clinically active macrovascular complications were excluded. The full inclusion and exclusion criteria are presented in the Supplementary Material. At the decision of the Sponsor, specific inclusion/exclusion criteria were waived on a case-by-case basis due to the impact of the COVID-19 global pandemic on patient recruitment rate.

### Ethics

The preclinical study protocol was approved by the local IACUC (Institutional Animal Care and Use Committee). The study was done in compliance with animal use guidelines, ARRIVE reporting, and ethical approval.

For the clinical study, all participants were required to provide written informed consent before undergoing screening procedures. Institutional ethical review board approval was obtained at each of the study sites before initiation. The relevant review board approval numbers are: University Health Network Research Ethics Board 19–5044, Ottawa Health Science Network Research Ethics Board (OHSN-REB) 20190419, Hamilton Integrated Research Ethics Board 8101 (McMaster University), University of Alberta (Alberta Diabetes institute) Pro00091282, Comité d’éthique de la recherche du CIUSSS de l’Estrie- CHUS 2020–3520. The study was conducted in accordance with Good Clinical Practice guidelines and complied with ethical standards described in the Declaration of Helsinki.

Central regulatory approval was received by Health Canada (Control number: 226063). The trial is registered with ClinicalTrials.gov NCT04005287.

### Randomisation and masking

Participants with DPN were randomised to 1 of 4 treatment groups in a 1:1:4:4 ratio as follows: placebo control 2 mL (n = 6 subjects); placebo control 4 mL (n = 6 subjects); low dose pirenzepine (2 mL, 73 mg pirenzepine free base monohydrate) (n = 24 subjects); and high dose pirenzepine (4 mL, 146 mg pirenzepine free base monohydrate) (n = 24 subjects). Randomisation was performed using an interactive web-based response system that randomly assigned treatment groups to subjects eligible for recruitment. The sponsors, investigators and participants were unaware of treatment allocations during the study treatment and follow-up periods. Blinded clinical and safety assessments were done by the investigators and the sponsor conducted the blinded safety reviews.

### Procedures

The study comprised a screening period (days −45 to day −28) during which participant eligibility was determined based on medical history, physical examination, review of entry criteria and IENFD score. Subsequently, the eligible participants were randomised at the baseline visit and entered a 24-week double-blind treatment period which included visits for efficacy and pharmacokinetic measurements at weeks 2, 4, 8, 12, 16, 20, and 24. Assessments for adverse events (AEs) and queries for concomitant medication usage occurred throughout the treatment period. At the post-treatment follow-up visit at week 26, participants were assessed for adverse events and queried for concomitant medication usage.

WST-057 (active ingredient: pirenzepine free base monohydrate, manufactured by Archimica S.p.A, Italy and study drug manufactured by Tergus Pharma, Durham NC, USA) or matching placebo was provided to participants in a labelled, airless metred-dose pump (140 g maximum fill, set to deliver 1 mL per pump) made of medical-grade polypropylene plastic on Study Day 1 and at Weeks 4, 8, 12, 16, and 20. The participants were instructed to self-administer the study drug by dispensing 1 or 2 pumps of investigational product into the palm of their hand and spread it evenly over each leg (from mid-calf to the sole). After application, participants were directed to allow the material to dry, prior to covering the dosing area with socks or clothing, and were reminded to apply the study drug at approximately the same time every day.

### Assessments at screening and 24 weeks

IENFD was evaluated at screening and at Week 24 via a skin punch biopsy. The screening or baseline punch biopsy was collected at least 28 days prior to Study Day 1 to facilitate biopsy site healing before the first dose of study drug. The skin punch biopsies were performed near the ankle, approximately 10 cm above the lateral malleolus and 3 cm to the posterior: and in the thigh approximately 25 cm below the iliac spine, 5 cm below the level of the pubis, and 8 cm to the posterior, using a 3 mm disposable punch under sterile technique after administration of topical anaesthesia with lidocaine. The end-of-study biopsy was collected no less than 2 cm and no more than 3.5 cm from the initial biopsy site from the same leg as the screening biopsies. Nerves present in the stained tissue were counted at a central laboratory (Calcutt Lab, UCSD) by a qualified, double-blinded observer as per established Lauria guidelines.^24^

### Assessments at screening, baseline, and 24 weeks

Quantitative thermal thresholds (cooling detection thresholds) were measured using the TSA-II Neurosensory Analyser (Medoc Advanced Medical Systems, Ramat-Yishai, Israel), using the method of limits, which is a psychophysical procedure for determining the sensory threshold by gradually increasing or decreasing the magnitude of the stimulus presented in discrete steps. The average of the 3 levels was calculated and reported for both feet. Vibration perception threshold testing was done using the Neurothesiometer (Horwell Scientific, London, UK), using the method of limits. Three measurements were recorded at each stimulation site and the average was reported for both feet. Sural nerve conduction velocity (SNCV) in m/s and a sural nerve amplitude in μV were recorded for each leg.

### Assessments at screening, baseline, and 2, 4, 8, 12, 16, 20, and 24 weeks

The disease assessment scales included the Norfolk Quality of Life Questionnaire-Diabetic Neuropathy (Norfolk QOL-DN), Toronto Clinical Neuropathy Score (TCNS) and the Modified Toronto Clinical Neuropathy Score (mTCNS). The Norfolk- QOL-DN is a comprehensive and validated 35-item questionnaire that captures the entire spectrum of DPN related to multiple domains such as activities of daily living, symptoms, large fibre neuropathy and physical functioning, small fibre neuropathy, and autonomic neuropathy.^25^ The pain assessment was performed using the visual analogue scale (VAS). The VAS was administered via a mobile device application, which prompted the subject to input their level of pain at the screening visit and on a weekly basis thereafter until week 24 or study termination.

### Safety assessments

Safety was monitored throughout the study. The safety assessments included adverse events, vital signs, physical examination, body mass index (BMI), clinical laboratory tests, 12-lead electrocardiogram (ECG), concomitant medications, and local skin irritation assessment using the Dermal Draize Scale.

### Pharmacokinetics

Trough plasma concentrations for pirenzepine and its desmethyl metabolite were collected at pre-specified time points (Weeks 2, 4, 8, 12, 16, 20, and 24). Testing was done using liquid chromatography coupled to tandem mass spectrometry to analyse mean and median concentrations per dose level per visit by Intertek Pharmaceutical Services, San Diego, CA USA. The LC-MS/MS method was validated with a dynamic range of 0.100–100 ng/mL. This bioanalytical partial method validation report describes the results of the analysis of pirenzepine in human plasma (K2EDTA). A summary of the validation data for pirenzepine is presented in the Supplementary Material (Supplementary Table S1).

### Outcomes

The primary objective of this study was to determine the safety and tolerability of 2 dose levels of WST-057 (4% pirenzepine free base monohydrate solution) after once-daily topical administration in participants with type 2 diabetes mellitus (T2DM) with peripheral neuropathy. The primary efficacy outcome measures of the change from baseline to Week 24 in the IENFD (ankle) and the impact on quality-of-life measures using the Norfolk-QOL-DN (total and the subset scores) were chosen based on the anticipated translation of the preclinical studies, but were not powered to achieve statistical significance in this early proof-of-concept study. The secondary efficacy outcomes included changes from baseline to Week 24 in the quantitative thermal threshold, quantitative vibration perception threshold, Toronto Clinical Neuropathy Score (TCNS), modified Toronto Clinical Neuropathy Score (mTCNS) visual analogue-scale (VAS) for pain, and the change in IENFD in an untreated location (thigh, at Week 24).

Adverse events were monitored from study entry until the follow up visit at 2 weeks after the last dose (Study Week 26). Safety events included reports of treatment-emergent adverse events and serious adverse events. The adverse events were coded using Medical Dictionary for Regulatory Activities (MedDRA) version 22.0.

### Statistics

For preclinical studies, the study design and analysis plan were agreed a priori. Raw data are not currently available for distribution. Processed data are presented as group mean ± SEM with between-group comparisons by one way ANOVA. Where Bartlett's test confirmed equal variances across the three groups (p > 0.05) the Holm-Sidak post-hoc test was used to identify differences between groups (Prism, Graphpad Inc, CA, USA).

For clinical studies, the population size and duration of the study was based on a similar phase-2 study designed to observe a positive change in the Norfolk-QOL-DN measurement in 60 subjects with type 2 diabetes mellitus after 6 months treatment with topiramate and ruboxistaurin.^26^ No formal power calculations were performed to estimate appropriate group sizes since this was the first clinical evaluation of pirenzepine in DPN. Cohort size was selected to achieve an appropriate balance between unnecessarily exposing many subjects to a novel therapeutic regimen, having sufficient subjects enrolled to interpret pharmacodynamic data, and making a reasonable assessment of the dose-related safety.

The primary objectives of the clinical trial were for the evaluation of safety and to meet the pilot trial objectives for efficacy to inform future design of a phase 3 trial if the Phase 2a study results are supportive. From this perspective, the a priori statistical analysis for the IENFD and QoL secondary outcomes were to compare the within-group baseline to 24-week changes by one-way ANOVA in a pilot design. The analytical populations were determined prior to database lock: the safety population consisted of all subjects who received at least one dose of study drug; the modified-intent-to-treat (mITT) population included all subjects randomised, who had at least one post-baseline measurement.; and the per protocol (PP) population was made up of mITT subjects with >80% self-reported compliance without major protocol deviations, such that they could aid in a better understanding of the impact of the study treatment, when administered as specified in the protocol. The list of the PP subjects was finalised by the sponsor prior to unblinding of the database, to avoid selective reporting. No formal interim analysis was planned for this study. All descriptive statistical analyses were performed using SAS statistical software (Version 9.4 or higher), unless otherwise noted.

Medical History and adverse events were coded using the Medical Dictionary for Regulatory Activities (MedDRA) version 22.0. Concomitant medications were coded using the World Health Organisation Drug Dictionary Enhanced (WHO-DDE, MAR2019). Demographic and baseline parameters were compared between treatment groups using descriptive statistics. Means (standard deviations) were calculated for continuous variables and numbers (percentages) were calculated for categorical variables. ANCOVA and mixed models for repeated measures (MMRM) were used to detect statistically significant differences in means among the treatment groups. P < 0.05 was considered significant. P-values are considered informative not for testing.

### Role of funders

The CIHR and Research Manitoba had no role in study design, data collection, data analyses or writing the report. WinSanTor personnel performed the preclinical study and helped with the clinical study design and data collection, performed the data analyses, and were involved in the review process (interpretation). WinSanTor did not write the report. All authors had full access to all the data in the study and the corresponding author had the final responsibility for the decision to submit for publication.

## Results

**Fig. 1:**
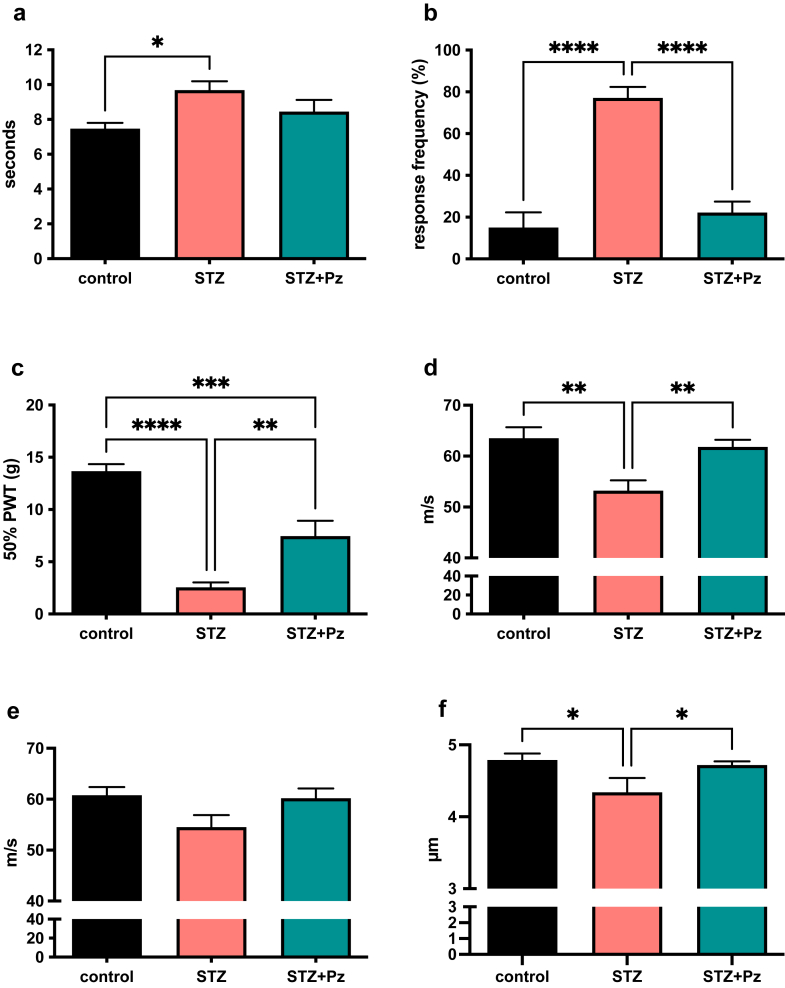
**Efficacy of topical pirenzepine formulation against neuropathy in rats.** STZ-diabetic rats were treated with 100 μl of 4% pirenzepine cream (STZ + Pz: blue bars) to the hind paw for 6 weeks before comparison of paw heat response latency (a), cold response frequency (b), response to von Frey filaments (c), motor (d) and sensory (e) nerve conduction velocity and tibial nerve mean axonal diameter (f) against values from control rats (black bars) and vehicle-treated STZ-diabetic rats (red bars). Data are group mean + SEM. For all panels the group sizes are control N = 8, diabetic + vehicle N = 7, diabetic + pirenzepine N = 9. Statistical comparisons by one way ANOVA with the Holm- Sidak post hoc test.∗ = p < 0.05,∗∗ = p < 0.01,∗∗∗ = p < 0.001,∗∗∗∗ = p < 0.0001.

No diabetic rats required supplemental insulin treatment and no adverse events were noted in the rats. One rat in the diabetes + vehicle group was removed from the analysis as it was not hyperglycaemic (designated a priori as being a blood glucose concentration of ≥270 mg/dl) at study end. For all reported data the group sizes are therefore: control N = 8; diabetic + vehicle N = 7; diabetic + pirenzepine N = 9. At study end, diabetic rats were significantly (p < 0.001) hyperglycaemic compared to age-matched controls (108 ± 8 mg/dl), whether treated with vehicle (594 ± 13) or pirenzepine (562 ± 11). Body weight was similar across control rats (292 ± 4 g) and diabetic rats treated with vehicle (298 ± 12) or pirenzepine (284 ± 10). Vehicle treated diabetic rats showed significant heat hypoalgesia, cold hyperalgesia, tactile allodynia and nerve conduction velocity slowing, all of which were prevented or attenuated in diabetic rats treated with 4% pirenzepine (Fig. 1A–E). Mean diameter of myelinated axons was significantly (p < 0.05) reduced in tibial nerve of diabetic rats and this was prevented by pirenzepine treatment (Fig. 1F), while the g-ratio was similar in all groups (control + vehicle = 0.595 ± 0.004, diabetic + vehicle=0.586 ± 0.016, diabetic + pirenzepine = 0.593 ±0.007).

**Fig. 2:**
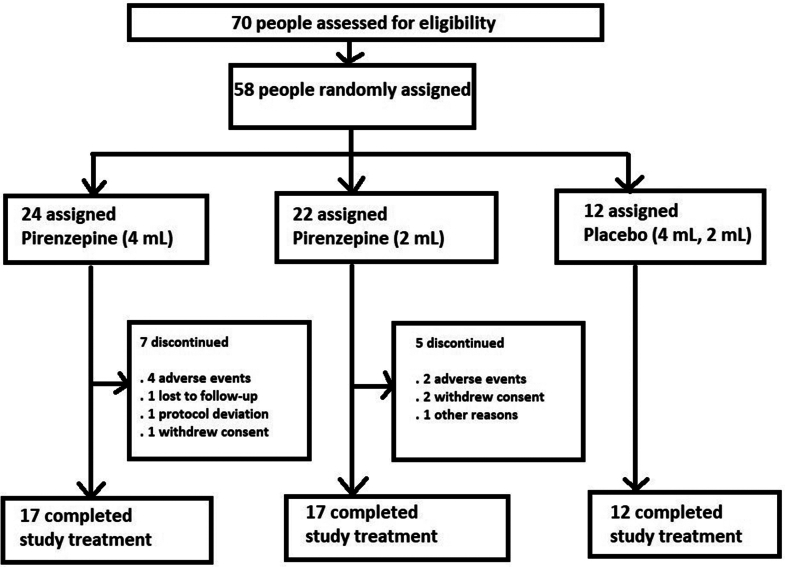
Trial profile.

Seventy participants were screened for the human study, of whom 12 were not randomised (Fig. 2). The first patient was screened on Oct 24, 2019. Fifty-eight participants were randomly assigned to pirenzepine 4 mL (n = 24), pirenzepine 2 mL (n = 22) or placebo (4 mL and 2 mL, 6 in each group). All 58 participants who were randomised were included in the safety analysis set, 57 of whom were also included in the mITT analysis set. Only 35 participants from the mITT group were eligible for the PPP due to early withdrawal (n = 10); major protocol violations (n = 8) and compliance violations (n = 2).

Forty-six of the 58 participants (79.3%) completed the study treatment period of 24 weeks. Seven participants (29.2%) in the pirenzepine 4 mL group and five in the pirenzepine 2 mL group (22.7%) discontinued the study. All subjects in the placebo group completed the study treatment. The reasons for discontinuation included adverse events,^5^ subject withdrawal^2^ and protocol deviation,^27^ loss to follow-up^27^ and other reason (moving to new province, 1) (Fig. 2). Of the total 580 cumulative visits scheduled, 109 (18.7%) were impacted by the Covid pandemic.

The demographic characteristics and clinical features were similar between the treatment groups (Table 1). The majority were male (38, 66.7%) and 24 (42.1%) were above the age of 65 years. Forty-three (75.4%) were white and 44 (77.1%) were on medications for neuropathic pain. The baseline severity of clinical involvement as assessed by parameters including baseline IENFD (ankle, thigh), Norfolk-QOL-DN total score, sural nerve conduction velocities and SNAP amplitudes, quantitative cooling thresholds, quantitative vibration perception thresholds, TCNS, mTCNS and VAS were similar between the study treatment groups. At baseline there were no meaningful demographic differences between the dose groups. Using shift analysis analysing baseline to Week 24, there were no trends or meaningful changes identified in vital signs (i.e., systolic and diastolic blood pressure, heart rate, respiratory rate, body mass index), ECGs or safety labs (i.e., glucose, TRIG, etc). While HbA1c data was not collected in this study of pirenzepine, in a similar study of the M1 antagonist, oxybutynin, also in a T2DM DPN population, no changes in HbA1c were detected after 20 weeks of topical dosing.^28^

**Table 1:** Baseline and demographic data comparison between the study treatment groups.

	Pirenzepine 4 mL (n = 24)	Pirenzepine 2 mL (n = 21)	Placebo (n = 12)
Age (years)	63 (8.1)	64 (6.1)	60.9 (8.6)
Age category >65 years	12 (50%)	9 (42.9%)	3 (25%)
Male	16 (66.7%)	14 (66.7%)	8 (66.7%)
Female	8 (33.3%)	7 (33.3%)	4 (33.3%)
Race			
White	18 (75%)	15 (71.4%)	10 (83.3%)
Asian	3 (12.5%)	2 (9.5%)	1 (8.3%)
African American	3 (12.5%)	1 (4.8%)	1 (8.3%)
Others		3 (14.3%)	
Body mass index (kg/m^2^)	33.6 (7.2)	30.5 (5.7)	36.7 (10.6)
Concomitant medicines for neuropathic pain	17 (70.8%)	18 (85.7%)	9 (75%)
Pregabalin/Gabapentin	10 (41.6%)	13 (59.1%)	5 (41.6%)
Amitriptyline/nortriptyline	2 (8.3%)	2 (9.1%)	1 (8.3%)
Duloxetine	5 (20.8%)	1 (4.5%)	–
Escitalopram	–	2 (9.1%)	1 (8.3%)
IENFD baseline ankle (IENF/mm)	3.9 (2.9)	4.2 (2.9)	3.3 (2.6)
Quality of life (Norfolk-QOL-DN)	36.1 (26.1)	44.4 (28.4)	29.8 (23.9)
Sural nerve conduction velocity (metres/sec)			
Average	36.8 (10.2)	39 (10.3)	33.3 (11.5)
Left leg	36.6 (10.1)	38.6 (10.2)	33.4 (11.5)
Right leg	37.1 (10.5)	39.3 (10.7)	33.3 (11.5)
Sural SNAP amplitude (microvolt) Average	4.1 (2.6)	4.2 (2.9)	3.1 (2.2)
Quantitative thermal threshold (Celsius) Average	22.6 (5.7)	24.3 (5.3)	22.8 (5.3)
Quantitative vibration threshold (volts) Average	59 (58.2)	27.9 (22.7)	52.5 (52.4)
Toronto Clinical Neuropathy score Total score	10.1 (2.8)	9.3 (2.8)	11.9 (3.1)
Modified Toronto Clinical Neuropathy score	14.8 (7.2)	14.8 (6.7)	14.4 (6.5)
Visual analogue scale (Average assessment value)	36.3 (22.1)	36.7 (25.7)	21.8 (24.6)
IENFD distant site (thigh) (IENF/mm)	6.6 (3.9)	7.0 (4.6)	6.7 (3.8)

Data are presented as mean (SD) or number (percentage). IENFD: Intraepidermal nerve fibre density, Norfolk-QOL-DN: Norfolk Quality of Life-Diabetic Neuropathy, SNAP: sensory nerve action potential.

An overview of the adverse events is presented in Table 2. Seventeen (70.8%), 16 (72.7%) and 12 (100%) subjects in the pirenzepine 4 mL, pirenzepine 2 mL and placebo group respectively reported at least one adverse event during the study. There were no deaths in any cohort. There were two serious adverse events reported in one subject in the 4 mL group: hyperkalemia and angina pectoris which was considered unrelated to study drug. Participants with adverse events with possible/probable/definite relation to study drug were 12 (50%) and 8 (36.3%) in the pirenzepine 4 mL and pirenzepine 2 mL groups respectively and 5 (41.7%) in the placebo. None of the subjects in any cohort had any moderate or severe skin changes; all the grades reported in the individual components of the Dermal Draize Score (dryness, erythema, pruritus, burning/stinging and oedema) were less than two. There were no neurological adverse events reported at the 4 mL group; in the pirenzepine 2 mL group six events were reported including headache (3, 13.6%) and dizziness, carpal tunnel syndrome and cerebrovascular accident one each (4.5%) and placebo (2, 16.7%, one each with burning and paraesthesias). Blurred vision was reported in one subject (pirenzepine 2 mL group). Mild neutrophilic leucocytosis was reported in four subjects (two each in pirenzepine 4 mL and 2 mL groups) and mild transaminitis in one (pirenzepine 4 mL group). There were no significant differences in the overall number and severity of treatment-emergent systemic adverse events between the treatment groups. However, treatment related bilateral skin rash was responsible for the early withdrawal or termination of four participants in the 4 mL dose group and two participants in the 2 mL and no patients in the placebo group.

**Table 2:** Adverse events.

Event	Pirenzapine 4 mL N = 24	Pirenzapine 2 mL N = 22	Placebo N = 12	p-Value 4 mL	p-Value 2 mL	p-Value Placebo
Any AE	17 (70.8)	16 (72.7)	12 (100.0)	0.070	0.133	0.053
Death	0	0	0			
Serious and severe AE	1 (4.2) angina pectoris	0	0			
TEAE-R						
None	5 (20.8)	7 (31.8)	2 (16.7)	1.00	0.429	0.710
Improbable	0	1 (4.5)	5 (41.7)	0.002	0.016	0.001
Possible	3 (12.5)	4 (18.2)	3 (25.0)	0.378	0.686	0.424
Probable	7 (29.2)	3 (13.6)	2 (16.7)	0.685	1.000	1.000
Definite	2 (8.3)	1 (4.5)	0	0.543	1.000	1.000
TEAE Severity						
Mild	6 (25.0)	8 (36.4)	5 (41.7)	0.446	1.000	0.509
Moderate	11 (45.8)	7 (31.8%)	7 (58.3)	0.725	0.273	0.332
Severe	0 (0.0)	1 (4.5)	0 (0.0)		1.000	1.000
TEAE-SD	6 (25.0)	3 (13.6)	0 (0.0)	0.079	0.284	0.180
TEAE						
Site Reactions	12 (50)	7 (31.8)	2 (16.7)			
MSK and CT	3 (12.5)	4 (18.2)	4 (33.5)			
Infection & Infestation	5 (20.8)	4 (18.2)	0			
GI Disorders	3 (12.5)	2 (9.1)	3 (925.0)			
Nervous System Disorders	0 (0.0)	6 (27.3)	2 (16.7)			
Influenza-like illness	0 (0.0)	2 (9.1)	0 (0.0)			
Injury	2 (8.3)	1 (4.5)	3 (25.0)			
Leucocytosis	2 (8.3)	2 (9.1)	0 (0.0)			
Transaminitis	1 (4.2)	0 (0.0)	0 (0.0)			

Data as number (%), MITT Analyses. The p values were calculated using exact methods and compared against placebo.

AE = adverse event, TEAE = treatment emergent adverse event, TEAE-R = treatment emergent adverse event as related to study drug.

TEAE-SD = treatment emergent adverse event leading to study discontinuation.

MSK and CT = musculoskeletal and connective tissue disorders.

GI Disorders = gastrointestinal disorders.

The Sponsor consulted a dermatologist after five subjects had already discontinued from the trial due to bilateral rash. Based on de-identified pictures of several of the withdrawn patients, a diagnosis of eczema craquele or asteatotic eczema was made. Eczema craquele is a dermatological condition that is characterised by dry, fissured skin that occurs from epidermal water loss. To treat this condition, the dermatologist prescribed a high-grade moisturising cream or ointment for daily topical use, which was provided to patients. After the initiation of the moisturiser, there were no additional early terminations due to rash. Contributing factors to the higher incidence of bilateral rash requiring withdrawal, could be related to the lower percent of emollient and the higher concentration of ethanol in the active formulation, when compared to placebo, which removes the natural oils from the skin; and that the 4 mL dose level was twice the volume of the 2 mL dose level (explaining the higher incidence in the 4 mL group versus the 2 mL group). As eczema craquele is caused by and exacerbated by drying agents, a formulation higher in ethanol and lower in emollient would result in exaggerated drying action. The improvement in associated topical adverse events after the introduction of daily use of high-grade moisturiser further supports the diagnosis of the eczema craquele, as opposed to local toxicity. As a result of these findings, future formulations for the active and the placebo will be equivalent in emollient to ethanol ratio, such that the drying action will be the same across dose groups and high-grade moisturiser will be provided.

There were no significant changes in concomitant medications, vital signs (including BMI), laboratory values or ECGs as reported by the baseline to Week 24 shift tables.

**Fig. 3:**
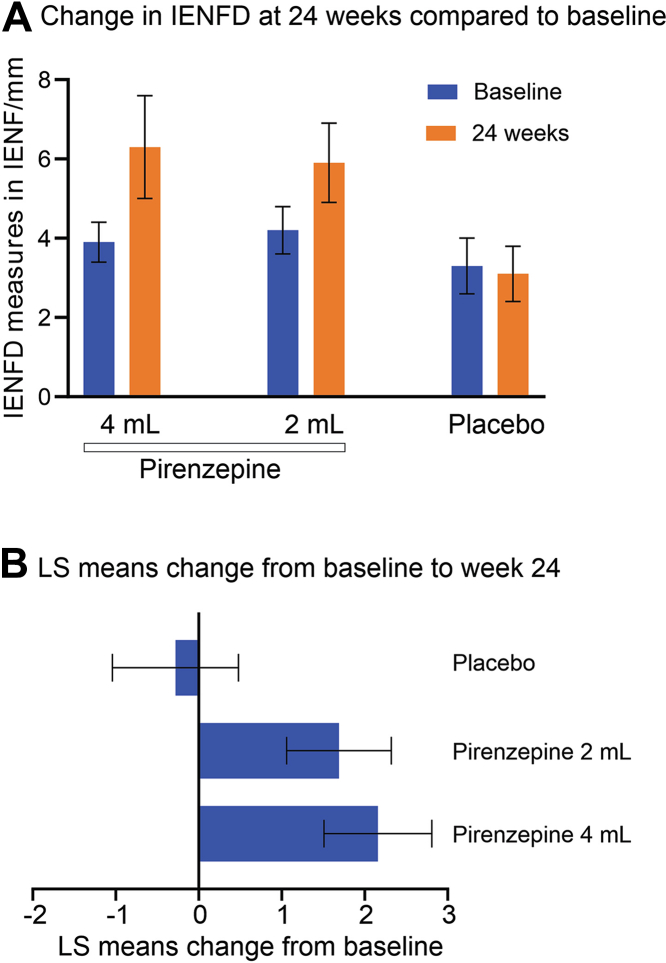
**Changes in intraepidermal nerve fibre density (IENFD) at the ankle in human participants.** (a)-Change in the mean IENFD (ankle) in the three groups at 24 weeks compared to baseline, and (b)-LS means change in IEND at 24 weeks compared to baseline. For both panels the group sizes are placebo (N = 12), pirenzepine 4 mL (N = 24), pirenzepine 2 mL (N = 21).

Fig. 3 (A) shows mean values for IENFD at baseline and study end (24 weeks) for the placebo and pirenzepine treatment groups. The least squares (LS) mean in change from baseline to week 24 in the IENFD at the ankle was 2.32 (p = 0.006, ANCOVA) in the pirenzepine 4 mL group; 1.50 (p = 0.048, ANCOVA) in the pirenzepine 2 mL group and −0.71 (p = 0.39, ANCOVA) in the placebo group (Fig. 3B). The change in IENFD was significant in the pirenzepine groups compared to placebo (p = 0.012, ANCOVA). The change was significant in the pirenzepine 4 mL group compared to placebo (p = 0.013, ANCOVA). The differences between pirenzepine 2 mL group compared to placebo did not reach significance (p = 0.054).

**Fig. 4:**
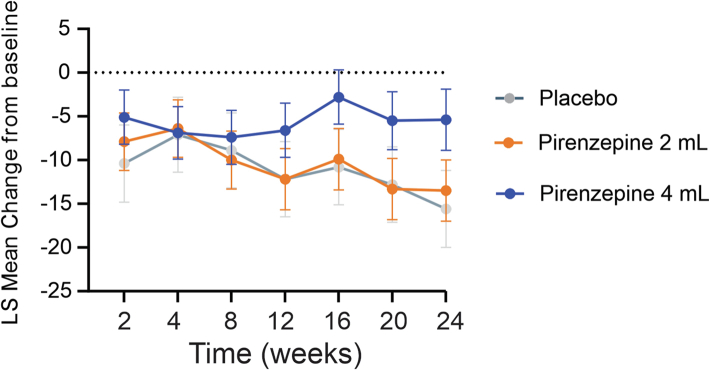
Change in the LS means of the Norfolk-QOL-DN total score from baseline to week 24 in the three groups. The group sizes are placebo (N = 12), pirenzepine 4 mL (N = 24), pirenzepine 2 mL (N = 21).

The change from baseline to Week 24 in the Norfolk-QOL-DN total score did not significantly differ between the treatment groups in the mITT analysis set (Table 3). However, using a mixed model for repeated measures (MMRM) in the PP analysis set, the LS mean change from Baseline to Week 24 in the Norfolk-QOL-DN total score was −6.9 (p = 0.06, MMRM) in the pirenzepine 4 mL group; −14.2 (p < 0.001, MMRM) in the pirenzepine 2 mL group and −10.6 (p < 0.001, MMRM) in the combined pirenzepine 4 mL and 2 mL groups (Fig. 4). The corresponding LS mean change from Baseline to Week 24 in the placebo group was −4.3 (p = 0.38. MMRM). When combining both active dose groups (4 mL and 2 mL) in the PP population using MMRM analysis to analyse Total Norfolk Score, a highly significant (p < 0.001, MMRM) within patient improvement of 10.4 points over placebo was observed after 24 weeks of dosing (Table 4). No significant changes were noted in the Norfolk-QOL-DN subset scores for symptoms, activities of daily living, large fibre function, small fibre function and autonomic function in the mITT analysis (Table 3). However, MMRM analysis of the Physical Function and Large Fibre subset score of the PP population for both active doses combined resulted in a highly significant (p < 0.001, MMRM) 6.0-point within patient improvement over placebo after 24 weeks of dosing.

**Table 3:** Comparison in efficacy outcomes between the treatment groups (MITT analysis).

Change at 24 weeks compared to baseline mean (SD)	Pirenzepine 4 mL N = 24	Pirenzepine 2 mL N = 21	Total Pirenzepine 4 mL and 2 mL, N = 45	Placebo N = 12
IENFD at application site (ankle, IENF/mm)	2.3 (3.6)	1.5 (2.0)	1.8 (2.8)	−0.6 (2.4)
Norfolk QOL-DN Total Score (reduction in score means improvement in clinical status)	−5.5 (9.6)	−15.4 (21.5)	−10.4 (17.2)	−13.9 (24.2)
Symptom score	−2.5 (4.5)	−1.3 (5.0)	−1.9 (4.7)	−2.8 (5.2)
ADL score	−0.3 (1.8)	−0.6 (3.1)	−0.4 (2.5)	−1.6 (3.3)
Small fibre score	−0.2 (1.7)	−2.1 (3.1)	−1.1 (2.5)	−1.8 (3.3)
Physical functioning/large fibre score	−2.6 (5.9)	−10.5 (15.1)	−6.6 (12.0)	−7.8 (16.1)
Autonomic score	0.3 (1.5)	−0.8 (3.2)	−0.3 (2.5)	−0.4 (2.8)
Modified Toronto Clinical Neuropathy Score—Total (reduction in score means improvement in clinical status)	−2.6 (4.7)	−4.7 (5.7)	−3.7 (5.3)	−4.7 (8.5)
Toronto Clinical Neuropathy Score—Total (reduction inscore means improvement in clinical status)	−0.5 (2.0)	−1.2 (4.2)	−0.9 (3.2)	−2.3 (2.6)
Quantitative cooling detection threshold (Celsius, increment means improvement)	1 (5.7)	−0.5 (2.0)	0.3 (4.3)	−0.5 (3.1)
Quantitative vibration perception threshold (volts, increment means worsening)	5.2 (19.9)	4.8 (20.7)	5.0 (20.0)	7.4 (25.5)
Sural nerve conduction velocity in meters/second (increment means improvement) Average	1.8 (6.0)	2.7 (6.1)	2.2 (6.0)	0.4 (2.5)
Sural SNAP (microvolt) (Reduction means worsening) Average	0.4 (1.1)	0.3 (2.4)	0.3 (1.9)	−0.2 (1.1)
VAS (Reduction in score means better clinical status)	−8.9 (18.2)	−9.2 (25.3)	−9 (21.0)	−1.8 (17.3)
IENFD at distant site (thigh, IENF/mm)	0.3 (4.8)	2.7 (5.4)	1.6 (5.2)	−0.4 (4.2)

Several subjects in the mITT population had protocol deviations (PDs) which would have excluded them from trial (i.e., administration of new pain medication after study initiation, etc.). However, due to the COVID restrictions impacting the monitoring visits, these PD were not realised until after the subjects completed the study. As COVID also impacted the enrolment rate, these subjects were not able to be replaced.

Data are presented as mean (SD) or number (percentage). IENFD: Intraepidermal nerve fibre density, Norfolk-QOL-DN: Norfolk Quality of Life-Diabetic Neuropathy, ADL: activities of daily living, SNAP: sensory nerve action potential, VAS: visual analogue scale, PGIC: patient global impression of change.

**Table 4:** Comparison in efficacy outcomes between the treatment groups (PP analysis).

Change at 24 weeks compared to baseline Mean (SD)	Pirenzepine 4 mL	Pirenzepine 2 mL	Total Pirenzepine 4 mL and 2 mL	Placebo
IENFD at application site (ankle, IENF/mm)	1.8 (3.2)	1.7 (2.1)	1.8 (2.7)	−1.1 (2.6)
Norfolk QOL-DN Total Score (reduction in score means improvement in clinical status)	−7.7 (8.1)	−15.6 (23.9)	−11.5 (17.7)	−1.1 (8.7)
Symptom score	−3.4 (3.8)	−2 (5.4)	−2.8 (4.5)	0 (2.2)
ADL score	−0.4 (2.0)	−0.7 (3.6)	−0.6 (2.8)	−0.1 (1.4)
Small fibre score	−0.3 (1.8)	−1.8 (1.8)	−1 (1.9)	−0.1 (1.2)
Physical functioning/large fibre score	−3.7 (5.9)	−10.8 (16.4)	−7.1 (12.5)	−0.9 (8.5)
Autonomic score	0.3 (1.7)	−0.2 (2.1)	0.1 (1.9)	−0.3 (3.1)
Modified Toronto Clinical Neuropathy Score—Total (reduction in score means improvement in clinical status)	−2.9 (5.1)	−5.4 (5.8)	−4.2 (5.5)	−1.6 (5.4)
Toronto Clinical Neuropathy Score—Total (reduction in score means improvement in clinical status)	0.4 (2.1)	−1.0 (3.5)	−0.7 (2.8)	−1.5 (1.2)
Quantitative cooling detection threshold (Celsius, increment means improvement)	−0.2 (3.2)	−0.6 (2.1)	−0.4 (2.6)	−0.3 (3.7)
Quantitative vibration perception threshold (volts, increment means worsening)	6.1 (21.7)	7.9 (22.2)	7.0 (21.4)	−1.1 (16.3)
Sural nerve conduction velocity in meters/second (increment means improvement) Average	0.3 (4.0)	−0.1 (9.1)	0.1 (7.1)	0.7 (2.8)
Sural SNAP (microvolt) (Reduction means worsening) Average	0.5 (1.1)	0.6 (2.0)	0.6 (0.6)	−0.2 (1.3)
VAS (Reduction in score means better clinical status)	−10.2 (18.8)	−17.8 (19.0)	−13.6 (18.7)	0.8 (7.8)
IENFD at distant site (thigh, IENF/mm)	0.4 (4.7)	2.4 (5.5)	1.0 (5.2)	0.7 (3.6)

Data are presented as mean (SD) or number (percentage). IENFD: Intraepidermal nerve fibre density, Norfolk-QOL-DN: Norfolk Quality of Life-Diabetic Neuropathy, ADL: activities of daily living, SNAP: sensory nerve action potential, VAS: visual analogue scale, PGIC: patient global impression of change.

**Fig. 5:**
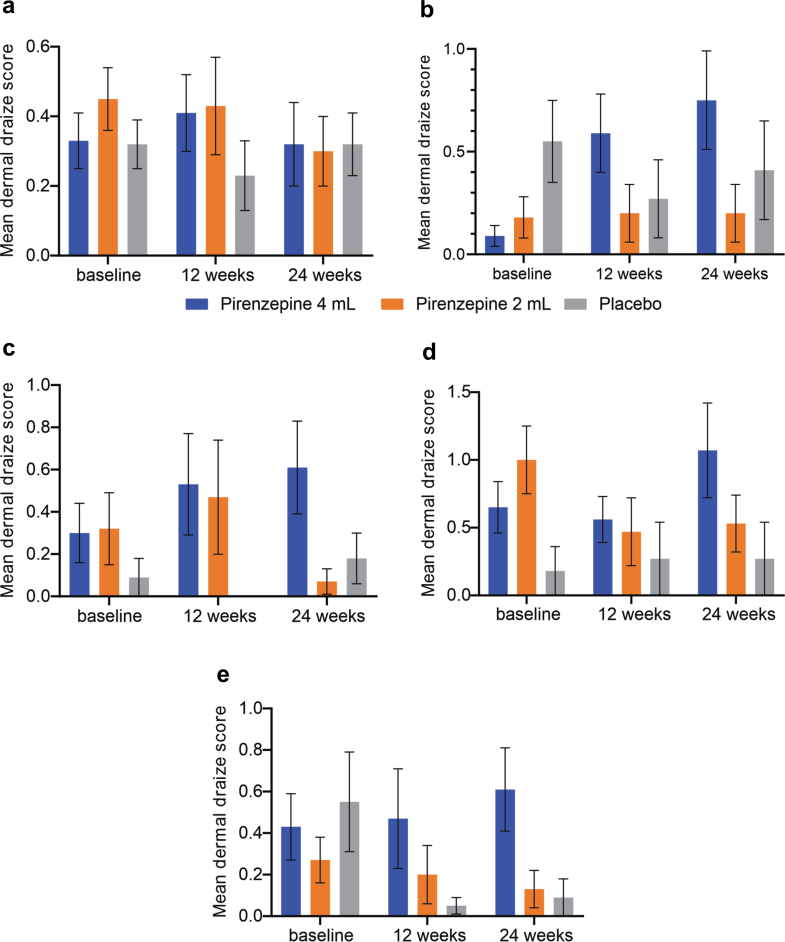
Mean Dermal Draize Score (individual components) comparison between the three groups at baseline, 12 weeks, and 24 weeks (individual components: (a) dryness, (b) erythema, (c) pruritus, (d) burning/stinging and (e) oedema). For all panels, the group sizes are placebo (N = 12), pirenzepine 4 mL (N = 24), pirenzepine 2 mL (N = 21).

No significant differences between the treatment groups were noted with respect to the changes from baseline to week 24 in the quantitative cooling detection thresholds, quantitative vibration perception thresholds, SNCV and SNAP amplitudes and change in patient global impression of change (Table 3). Of note, SNCV improved by 1.89 m/s in the combined (4 mL and 2 mL) active dose groups versus the placebo (Table 3). The change from baseline in the TCNS, mTCNS and VAS did not differ between the treatment groups as depicted in Table 3 and Fig. 5. There was a 2.6-point improvement in the mTCNS in the combined treatment group over placebo at week 24 in the PP analysis set (Table 4). There was no significant difference in the change of IENFD at a distant site (thigh), outside of the dosing area, from baseline to week 24 between the three groups.

**Fig. 6:**
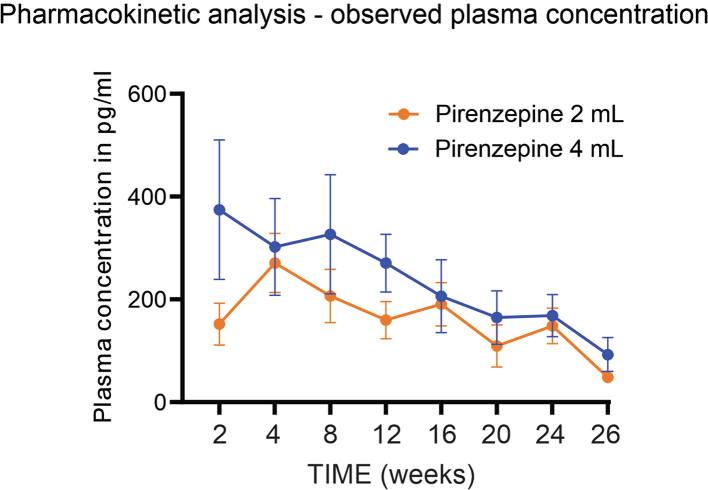
Pharmacokinetic analysis of the observed plasma concentration between the 4 mL and 2 mL groups. The group sizes are pirenzepine 4 mL (N = 24), pirenzepine 2 mL (N = 21).

The pharmacokinetic analysis shows a dose-dependent steady state concentration in the pirenzepine 4 mL and 2 mL groups with a gradual decline from week 2 until week 24. Mean trough plasma analysis for the 4 mL group ranged from 374.55 pg/mL at week 2 and decreased to 168.47 pg/mL at week 24; while the 2 mL group peaked at week 4 at 270.85 pg/mL and decreased 148.42 pg/mL by week 24 (Fig. 6). Trough concentration levels were roughly dose proportionate at week 2 but were increasingly comparable throughout the rest of the study. While only two participants were excluded due to poor compliance, the decreasing trough concentrations levels could indicate a reduced compliance over the 24-week trial; as well as explain the lack of dose response in some endpoints.

The 4 mL active dose is being taken forward into late-stage clinical trials, however, the formulation of the matching placebo will be revised such that both formulations contain the same amount of ethanol, which is thought to be the primary drying agent/contributor to the eczema craquele, as the high dose level proved to be equally as tolerable, when combined with a high-grade moisturiser, and it also demonstrated the highest nerve growth.

## Discussion

This phase 2a proof of concept clinical study successfully translated the nonclinical pharmacology and confirmed the proof of concept by meeting its primary efficacy outcome measure, showing a statistically significant increase in the IENFD at the ankle compared to baseline in the groups treated with pirenzepine (Fig. 3 and Table 3). In contrast, the placebo group showed a decline in IENFD, representing ongoing epidermal denervation in DPN over 6 months.

The major positive finding of the clinical study was that pirenzepine significantly elevated IENFD at the ankle, close to the site of drug application. This finding is consistent with a recent report of similar efficacy in subjects with diabetic neuropathy treated with the non-selective muscarinic antagonist oxybutynin^28^ and suggests M_1_R antagonism as the initial pharmacological approach to promote nerve regeneration in diabetic peripheral neuropathy. Prior pharmacological approaches including antioxidant,^29^ GLP-1 receptor agonists^30^ and mesenchymal stem cell-derived factors^31^ did not improve IENFD in diabetic subjects, while supervised exercise^32^ or simultaneous pancreas and kidney transplantation^33^ improved epidermal arborisation, but not regrowth from the dermis. It should be noted that there was no significant effect of pirenzepine at the thigh biopsy site, located more distant from the region of topical drug application (Table 3). The most likely reason is that topical drug application resulted in limited systemic exposure to pirenzepine as evidenced by the extremely low plasma concentrations. This is encouraging and consistent with the low level of drug-related systemic adverse side effects observed. An alternative rationale, not mutually exclusive, is that pirenzepine affords its growth promoting effect when acting locally at nerve endings in the skin. In mice with oxaliplatin-induced neuropathy topical application to the eye of muscarinic toxin 7 (MT7, the only specific negative allosteric modulator of M_1_R) protected corneal nerves and activated AMPK in the ipsilateral trigeminal ganglia, but not on the contralateral side.^14^ Conversely, studies in diabetic mice show that topical pirenzepine had neuroprotective effects distant to the site of application. The extent to which modifying WST-057 formulation and dosing to increase systemic levels of pirenzepine after topical application would expand the distribution of significant IENF regeneration requires further evaluation, along with consideration of other delivery methods that retain efficacy while minimising systemic side effects associated with antimuscarinics.

Over 45% of patients with T2DM and 54% of those with T1DM develop neuropathy.^34^ Despite this high prevalence of DPN there are no effective disease modifying pharmacotherapies to definitively treat the condition. Despite encouraging results from many preclinical studies, few interventions have demonstrated unequivocal evidence of efficacy and therapeutic benefit in large scale clinical trials and none have yet achieved approval by regulatory agencies. The rationale for the use of pirenzepine to treat DPN derives from preclinical studies demonstrating that diverse antimuscarinics prevent and reverse multiple functional and structural indices of peripheral neuropathy in rodent models of diabetes.^8^^,^^12^, ^13^, ^14^ Efficacy of antimuscarinics required specific or selective targeting of the M_1_R sub type and was associated with removal of tonic cholinergic suppression of mitochondrial respiratory capacity and neurite outgrowth.^8^^,^^11^ The proposed mechanisms of action include activation of AMP-activated protein kinase (AMPK) via a Ca^2+^-dependent pathway,^8^^,^^14^ augmentation of mitochondrial complex activity with increased expression of the respiratory electron transport chain components mediated via the AMPK pathway^8^ and modulation of cellular microtubule polymerisation through blockade of G protein signalling emanating from the M_1_R.^11^ Our selection of pirenzepine for clinical evaluation was driven by its relative selectivity of M_1_R antagonism, limited capacity to cross the blood brain barrier and established clinical safety profile. A topical route was chosen in light of the proven clinical effectiveness and favourable safety profile of topical preparations of other antimuscarinics^35^^,^^36^ and efficacy in our preclinical studies in diabetic rodents. Before proceeding with clinical studies, it was necessary to ensure that the formulation developed for topical delivery of pirenzepine to humans did not modify its efficacy. The clinical formulation of topical pirenzepine when applied to type 1 diabetic rats reproduced efficacy observed in preclinical studies using models of both type 1 and type 2 diabetes where pirenzepine was delivered in hydrogel. This supported advancing the formulation to clinical studies. Efficacy also extended to novel measures of cold hyperalgesia and reduced axonal diameter. The latter is particularly noteworthy as it offers a novel structural correlate to efficacy against large fibre conduction slowing and emphasises that antimuscarinics have the capacity to protect against functional and structural features of both large and small fibre neuropathy. The absence of change in g-ratio indicates that increased axonal diameter was not a consequence of pathological axonal swelling.

Other relevant clinical efficacy observations did not change in the mITT analysis set in contrast to published results with other agents where changes in quality of life and other neuropathy measures were observed.^26^ The current study showed positive, statistically significant effects in the pirenzepine groups compared to placebo included the positive improvement in both the total score in the Norfolk-QOL-DN score as well as the physical function large fibre subset score, as per the PP analysis. In the previous study, data on patient numbers completing the trial were not included in the publication, so we cannot assess potential bias in the outcomes. Further, it is unclear that topiramate use can be blinded successfully with an inactive placebo due to the sedating side-effects of the drug. While not statistically significant, SNCV in the (combined) active group (2 mL and 4 mL) improved by more than 1 m/s over placebo, a difference considered to be the minimal clinically important difference (MCID). The most plausible explanation for the observation that improvement in the IENFD at the ankle did not translate into more clinical benefits is that the current study was not adequately powered to analyse these end-points. Moreover, the beneficial clinical effects of increased epidermal reinnervation observed with pirenzepine may become more evident over a longer duration of study since the placebo group with untreated DPN is likely to have a progressive axonal degeneration.

There was no significant difference in the drug-related systemic adverse event profile on comparing the treatment and placebo groups. The majority of side effects were mild and any causal association with pirenzepine was uncertain. However, there were treatment-related application site skin reactions in 50% (pirenzepine 4 mL group), 31.8% (pirenzepine 2 mL group) and 16.7% in placebo treated subjects. The application site reactions were mainly in the form of erythema, rash, pruritus, and oedema. Five patients discontinued due to dermal topical side effects (4 at 4 mL and 1 at 2 mL pirenzepine). The vehicle was solvent based and appeared to be drying and irritating—particularly to the thin skin found in persons with diabetes. A higher volume (4 mL) resulted in a higher exposure to these solvents. Once a topical moisturiser was provided to the participants and its use integrated into their daily routine, no additional participants were withdrawn from the trial. None of the reactions were severe necessitating study drug interruption or discontinuation.

A major strength of this study is the novelty of demonstrating that efficacy of topical pirenzepine against indices of peripheral neuropathy in rodent models of diabetes translated into a significant effect on a biometric measure of small fibre pathology in subjects with type 2 diabetes and peripheral neuropathy. This indicates that preclinical efficacy studies in rodent models of type 1 and type 2 diabetes can predict clinical efficacy. The clinical study also represents the efficacy of topical pirenzepine in people with diabetic neuropathy. This is a major strength as there is no specific disease modifying therapy yet available for this complication of diabetes. The main limitation of the study was the number of protocol deviations due to the COVID-19 pandemic. As a result of emergency mandates including lockdowns, monitoring visits were significantly reduced resulting in a high number of protocol deviations specifically around inclusion/exclusion criteria. Under normal circumstances, participants with major deviations would have been screen failed, or should have been discontinued and replaced after being identified by the site or during a monitoring visit. However, during this unprecedented event, even if these individuals with major protocol deviations had been identified and early terminated in a timely manner, replacing them would have been very difficult. Due to the pandemic, enrolment suffered, as patients with diabetes were classified as a COVID-19-vulnerable population and many subjects were afraid to leave their homes and make hospital visits. Furthermore, the institutions conducting this study were under emergency conditions such that clinical trial enrolment, conduct, and follow-up were a lower priority. Another limitation is the quantification of IENFD by counting the number of fibres crossing the dermal:epidermal junction. While this approach is validated and widely used in clinical studies for detecting IENF loss in small fibre neuropathies,^24^ it does not incorporate any measure of terminal arborisation status and thus would not detect any effect of treatment on collateral sprouting in the epidermis. Being an early phase 2 study with a small sample size, the study was not powered for detecting statistical significance in the efficacy outcomes.

In conclusion, pirenzepine administered once daily for 24 weeks in patients with DPN, resulted in a significant increase in IENFD at the ankle, a primary efficacy outcome measure. The effect of this intervention was seen over a relatively short period of time. This observation is encouraging and has implications for other proof-of-concept studies in patients with DPN. The increase in IENFD was not accompanied by a statistically significant change in the clinical measures. However, the study was not specifically powered to detect functional recovery. Future studies with a larger sample size, preferably multi-centre and over a sufficiently longer duration of time will provide more robust evidence about the potential protective role of pirenzepine and similar muscarinic antagonists. Combining treatment strategies targeting different pathophysiological pathways and optimally addressing pain is an alternate option that may provide further insights in management of established DPN.

## Contributors

NAC, KEF, KG and AH designed, performed and analysed the preclinical study.

VB, PF, NAC, AH, AB, DWZ, NM, SG and BAP designed the clinical study. VB, AB, DWZ, NM, ZP and SG enrolled patients. AH did the statistical analysis. AS, VB, BAP, PF, NAC and AH developed the clinical study report. All authors accessed, verified, and interpreted the data. VB, PF and AH have directly assessed and verified the underlying data reported in the manuscript. All authors had full access to study data, reviewed, edited, and provided final approval of the manuscript content, and had final responsibility for the decision to submit for publication.

## Data sharing statement

The data for the preclinical and clinical studies are not available for sharing at this time pending patent approvals but will be made available upon reasonable request within a year of publication.

## Declaration of interests

PF and NAC are co-founders and shareholders in WinSanTor Inc. KEF and AH are employees and shareholders in WinSanTor Inc. VB is a consultant for Akcea Therapeutics, Inc.; Alexion Pharmaceuticals, Inc.; Alnylam Pharmaceuticals, Inc.; argenX; AstraZeneca; CSL; F. Hoffmann-La Roche Ltd; Grifols; Immunovant, Inc.; Ionis Pharmaceuticals; Janssen Global Services, LLC; Japan Tobacco; Johnson & Johnson Services, Inc.; Momenta; Novo Nordisk A/S; Octapharma USA, Inc.; Pfizer Inc.; Powell Mansfield Inc.; Sanofi; Takeda Pharmaceutical Company Limited. VB has received research support from Akcea Therapeutics, Inc.; Alexion Pharmaceuticals, Inc.; argenx; AstraZeneca; CSL; Grifols; Immunovant, Inc.; Ionis Pharmaceuticals; Johnson & Johnson Services, Inc.; Momenta; Octapharma USA, Inc.; Takeda Pharmaceutical Company Limited; UCB S.A. ZP has received honoraria for speaking and consulting from DexCom, Eli Lilly, NovoNordisk, Pfizer, Sanofi, and research grants from NovoNordisk. BAP received grants or contracts from NIH/NIDDK, Breakthrough T1D, Zucara pharmaceuticals, and Vertex Pharmaceuticals, and consulting fees from Abbott, Dexcom, Insulet, Novo Nordisk, Sanofi, Vertex and Nephris. BAP received payment or honoraria from Abbott, Dexcom, Insulet, Novo Nordisk, Bayer and Sanofi and participares in a data safety monitoring or advisory board for TINSAL-FISH Study (DSMB Chair). The rest of the authors do not have any relevant disclosures.
