# Supplementary material for: Topical application of the antimuscarinic pirenzepine increased lower limb nerve fibre density in a phase 2a study in type 2 patients with diabetes with peripheral neuropathy
Source: eBioMedicine. 2025 Dec 5;123:106055. doi: 10.1016/j.ebiom.2025.106055 (PMC12721300; doi:10.1016/j.ebiom.2025.106055)
Supplement: Supplementary Table [file mmc1.docx]

Supplementary Table 1. A summary of the validation data for pirenzepine.

| **Analyte:** | **Pirenzepine** |
| --- | --- |
| **Matrix:** | Human Plasma (K_2_EDTA) |
| **Internal Standard:** | Pirenzepine-d_8_ |
| **Analytical Procedure:** | LC-MS/MS |
| **Sample Preparation:** | Protein Precipitation with filtration |
| **Assay Aliquot Volume:** | 100 µL |
| **Assay Range:** | 0.100 – 100 ng/mL |
| **Regression:** | Quadratic Regression (1/x^2^) |
| **Validation Batch Acceptance** (Acceptable/Total Primary Runs): | 3/3 |
