# Supplementary material for: Topical application of the antimuscarinic pirenzepine increased lower limb nerve fibre density in a phase 2a study in type 2 patients with diabetes with peripheral neuropathy
Source: eBioMedicine. 2025 Dec 5;123:106055. doi: 10.1016/j.ebiom.2025.106055 (PMC12721300; doi:10.1016/j.ebiom.2025.106055)
Supplement: Supplementary Material 1 [file mmc2.pdf]

WINSANTOR

A Randomized, Double-Blind, Placebo-Controlled, Parallel, 24-Week, Phase 2a Study of Topical Pirenzepine (WST-057) or Placebo in Type 2 Diabetic Patients with Peripheral Neuropathy

**Protocol Number:**	WST-PZP-002
**Investigational Product:**	WST-057 (4% pirenzepine free base monohydrate)
**Development Phase:**	2a
**Sponsor Name and Address:**	WinSanTor, Inc.<br>7220 Trade St, #370<br>San Diego, CA 92121
**Protocol Date:**	April 16, 2019
**Protocol Version:**	2.0
**Amendment Version:**	3.0
**Amendment Date(s):**	January 01, 2021

CONFIDENTIALITY STATEMENT

The information in this document contains commercial information and trade secrets that are privileged or confidential and may not be disclosed unless such disclosure is required by applicable laws and regulations. In any event, persons to whom the information is disclosed must be informed that the information is privileged or confidential and may not be further disclosed by them. These restrictions on disclosure will apply equally to all future information supplied to you which is indicated as privileged or confidential.

INVESTIGATOR'S STATEMENT

I understand that all information concerning the product supplied to me by WinSanTor, Inc. (WinSanTor), in connection with this study and not previously published, is confidential information. This information includes the Investigator's Brochure (IB), protocol (and applicable amendments), Case Report Forms, assay methods, technical methodology, and basic scientific data.

I will conduct the study according to the protocol and I understand that any changes to the protocol must be approved in writing by WinSanTor and the Institutional Review Board (IRB) before implementation, except where necessary to eliminate apparent immediate hazards to the subjects.

I confirm that I will report all adverse events following the regulations referenced in the protocol.

I confirm that I will conduct this study in conformance with the principles of the Declaration of Helsinki, GCP, and United States law and regulations.

I confirm that I am informed of the need for records retention and that no data will be destroyed without the written consent of WinSanTor.

By my signature below, I hereby attest that I have read, understood, and agree to abide by all conditions, instructions, and restrictions contained in this protocol amendment dated January 01, 2021.

Investigator's Signature: ______________________________ ____________________
Date

Name (printed): ______________________________

1 PROTOCOL AMENDMENT CHANGES

Protocol Amendment Changes for Amendment 3:

Amendment 3: Dated 01 January 2021

Original Protocol, Version 2.0 dated 16 April 2019

Amendment 3 changes to the protocol WST-PZP-002 and the reasons for those changes are described below:

#	Protocol Change Description	Reason for Change
1	Updated the protocol version date	Updated date of issue for Amendment 3
2	Updated footer on the protocol with the new Amendment version and date	Updated for Amendment 3
3	Addition of the Patient Global Impression of Change to Week 24/EOS visit	Added per FDA recommendation
4	Included autoimmune conditions to Exclusion Criterion #29	To provide clarity for patient screening
5	Include palms of hands in the dermatological evaluation aspect of the physical exam	Added per FDA recommendation
6	Updated the address for a new PK laboratory (for bioanalytical sample analysis)	Previous lab went out of business
7	Updated the references per the new text for the Patients' Global Impression of Change	Literature References

SECTION NO.	ORIGINAL TEXT	AMENDMENT 1 TEXT CHANGES<br>~~STRIKE THROUGH~~ = DELETED TEXT<br>BOLD = INSERTED TEXT
**Cover Page**	Amendment Version 2.0	Amendment Version ~~2.0~~ **3.0**
**Footer on each page**	WinSanTor_WST-PZP-002-Amendment 2.0 01JUN2020	WinSanTor_WST-PZP-002-Amendment ~~2~~**3**.0 01~~JUN~~**JAN**202**1**
**Investigator's Statement**	By my signature below, I hereby attest that I have read, understood, and agree to abide by all conditions, instructions, and restrictions contained in this protocol amendment dated June 01, 2020.	By my signature below, I hereby attest that I have read, understood, and agree to abide by all conditions, instructions, and restrictions contained in this protocol amendment dated ~~June~~ **January** 01, 202~~0~~**1**.
**2 Abbreviations**		**PGIC Patient Global Impression of Change**
**4.3 Exploratory Objectives**	The exploratory objective evaluated in the study is to determine the impact (defined as change from baseline) of once-daily dosing of WST-057 on quantitative thermal threshold, quantitative vibration perception, visual analogue score (VAS), and the change in IENFD in an untreated location (thigh) at week 24	The exploratory objective evaluated in the study is to determine the impact (defined as change from baseline) of once-daily dosing of WST-057 on quantitative thermal threshold, quantitative vibration perception, visual analogue score (VAS), ~~and~~ the change in IENFD in an untreated location (thigh) at week 24 **and the Patients' Global Impression of Change (PGIC) at week 24/EOS.**
**4.4 Pharmacodynamic Endpoints**	…<br>• Visual Analogue Score<br>• Quantitative thermal threshold<br>• Quantitative vibration perception	…<br>• Visual Analogue Score<br>• Quantitative thermal threshold<br>• Quantitative vibration perception<br>• **Patients' Global Impression of Change**
**5.1 Study Design, End-of-study Procedures (Early Termination or Week 24 +3 days)**	…<br>• Quality of Life (Norfolk-QOL-DN) (week 24)<br>• Quantitative thermal threshold (week 24)<br>• Quantitative vibration perception (week 24)	…<br>• Quality of Life (Norfolk-QOL-DN) (week 24)<br>• Quantitative thermal threshold (week 24)<br>• Quantitative vibration perception (week 24)<br>• **Patients' Global Impression of Change (week 24**)
**5.3 Schedule of Assessments, Week 24/EOS**		**PGIC**
**6.3 Exclusion Criterion #29**	Pain or neuropathy from another cause (including central pain, radiculopathy, painful arthritis, etc.) at the discretion of the PI.	Pain or neuropathy from another cause (including central pain, radiculopathy, painful arthritis, **autoimmune and inflammatory diseases including rheumatoid arthritis, lupus, Sjogren's syndrome, vasculitic disorders such as periarteritis nodosa, Churg-Strauss, etc., celiac disease, Crohn's disease, ulcerative colitis, spondyloarthropathies, sarcoidosis,** etc.) at the discretion of the PI.
**7.1 Pharmacodynamic and Pharmacokinetic Evaluations**	7.1 Pharmacodynamic and Pharmacokinetic Evaluations<br><br>The pharmacodynamics of WST-057 will be assessed by evaluating the change from baseline in the following parameters:<br>• IENFD change from baseline for both treated and untreated skin (section 7.1.1)<br>• Quantitative thermal threshold (section 7.1.2)<br>• Quantitative vibration perception (section 7.1.2)	7.1 Pharmacodynamic and Pharmacokinetic Evaluations<br><br>The pharmacodynamics of WST-057 will be assessed by evaluating the change from baseline in the following parameters:<br>• IENFD change from baseline for both treated and untreated skin (section 7.1.1)<br>• Quantitative thermal threshold (section 7.1.2)<br>• Quantitative vibration perception (section 7.1.2)

SECTION NO.	ORIGINAL TEXT	AMENDMENT 1 TEXT CHANGES<br>~~STRIKE THROUGH~~ = DELETED TEXT<br>BOLD = INSERTED TEXT
		• **Patients' Global Impression of Change (section 7.1.2)**<br>•
**7.1.2 Patients' Global Impression of Change (PGIC):**		**…Patients' Global Impression of Change**<br><br>**The Patients' Global Impression of Change (PGIC) scale will be used to assess patients' perception of changes following treatment (i.e., "feeling better" or "feeling worse"). The PGIC is a 7-point verbal scale and is commonly used in clinical studies assessing pain relief following treatment, due to its ease of administration and scoring and because it is a generic scale applicable to a wide variety of conditions and treatments including diabetic peripheral neuropathy (Perrot and Lanteri-Minet,2018) It has been demonstrated that the subjective "much improved" and "very much improved" ratings indicate moderately important and substantial improvement (Dworkin et al, 2008).**<br><br>**In this study, the PGIC will be administered to the patients on paper by site personnel at Study Week 24/EOS**
**7.2.4 Physical Examination**	This evaluation will include examination of the following body systems: general appearance; eyes, ears, nose, and throat; head and neck; chest and lungs; cardiovascular; abdomen; musculoskeletal; lymphatic (head and neck); dermatological; neurological; and extremities.	This evaluation will include examination of the following body systems: general appearance; eyes, ears, nose, and throat; head and neck; chest and lungs; cardiovascular; abdomen; musculoskeletal; lymphatic (head and neck); dermatological **(including the palms of hands to evaluate any investigational product reaction)**; neurological; and extremities.
**7.6 Pharmacokinetic Sampling**	Intertek Group plc<br>Attn: Sample Accessioning/Kjerstina Gutierrez<br>10420 Wateridge Cir<br>San Diego, CA 92121<br>United States of America<br>Email: kjerstina.gutierrez@intertek.com<br>Telephone: +1.858.558.2599	~~Intertek Group plc~~<br>~~Attn: Sample Accessioning/Kjerstina Gutierrez~~<br>~~10420 Wateridge Cir~~<br>~~San Diego, CA 92121~~<br>~~United States of America~~<br>~~Email: kjerstina.gutierrez@intertek.com~~<br>~~Telephone: +1.858.558.2599~~<br>**Q2 Solutions**<br>**Attn: Valerie L Belcher**<br>**19 Brown Road**<br>**Ithaca, NY 14850**<br>**USA**<br>**valerie.belcher@q2labsolutions.com**<br>**Office: +1 607 442 4544**<br>**Mobile: +1 512 840 9961**

SECTION NO.	ORIGINAL TEXT	AMENDMENT 1 TEXT CHANGES ~~STRIKE THROUGH~~ = DELETED TEXT BOLD = INSERTED TEXT
**16 References**		**Dworkin RH, Turk DC, Wyrwich KW, et al. Interpreting the Clinical Importance of Treatment Outcomes in Chronic Pain Clinical Trials: IMMPACT Recommendations. The Journal of Pain. 2008; 9(2): 105-121.**<br><br>**Perrot S, Lanteri-Minet M. Patients' Global Impression of Change in the management of peripheral neuropathic pain: Clinical relevance and correlations in daily practice. European Journal of Pain. 2019;23:1117-1128.**

TABLE OF CONTENTS

Description **Page**

Investigator's Statement.....2
1 Protocol AMENDMENT CHANGES.....3
2 List of Abbreviations and Definition of Terms.....11
3 Introduction and Rationale for Dose Selection.....13
3.1 Background Information.....13
3.2 Rationale for Study and Dose Selection.....14
4 Study Objectives.....15
4.1 Primary Objective.....15
4.2 Secondary Objectives.....15
4.3 Exploratory Objectives.....15
4.4 Pharmacodynamic Endpoints.....15
4.5 Safety Endpoints.....16
5 Study Description.....17
5.1 Study Design.....17
5.2 Stopping Criteria.....20
5.3 Schedule of Assessments.....21
6 Subject Eligibility and Withdrawal Criteria.....24
6.1 Number of Subjects.....24
6.2 Inclusion Criteria.....24
6.3 Exclusion Criteria.....25
6.4 Withdrawal Criteria.....27
7 Study Procedures and Assessments.....29
7.1 Pharmacodynamic and Pharmacokinetic Evaluations.....29
7.1.1 Intraepidermal Nerve Fiber Density.....29
7.1.2 Quantitative Sensory Testing.....30
7.1.3 Disease Assessment Scales.....31
7.1.4 Pain Assessment Scale: Visual Analogue Score.....31
7.2 Safety Evaluations.....32

7.2.1 Adverse Events 32
7.2.2 Clinical Laboratory Tests....32
7.2.3 ECG and Vital Signs....33
7.2.4 Physical Examination 34
7.2.5 Local Skin Irritation Assessment (Dermal Draize Score) 34
7.2.6 Demographics and Medical History 34
7.2.7 Body Weight, Height, and Body Mass Index 34
7.3 Pharmacokinetic Evaluations....35
7.4 Data Monitoring Committee....35
7.5 Total Blood Volume 35
7.6 Pharmacokinetic Sampling 35
8 Conduct of Study....37
8.1 Study Blinding and Randomization....37
8.2 Subject Informed Consent....37
8.3 Study Visits....38
8.3.1 Scheduled Visits 38
8.3.2 Unscheduled Visits 39
8.4 General Restrictions....39
8.4.1 Fasting Treatment Periods 39
8.4.2 Smoking....39
8.4.3 Physical Exercise 39
8.4.4 Contraceptive Measures....39
8.5 Compliance with the Protocol....40
8.6 Termination of the Study 40
9 Study Medication 41
9.1 Description of Study Medication....41
9.2 Drug Packaging....42
9.3 Drug Labeling 42
9.4 Drug Storage 43
9.5 Dispensing and Administration of Treatment....43
9.6 Drug Accountability....44
9.7 Procedure for Unblinding 44

10 Concomitant Medication **45**
10.1 General Considerations 45
10.2 Prohibited Medication 45
10.3 Permitted Medication 45
11 Adverse Events **46**
11.1 Adverse Event Definition 46
11.2 Reporting of Adverse Events 46
11.3 Follow-Up of Adverse Events 48
12 Serious Adverse Events **49**
12.1 Life-threatening Adverse Event 49
12.2 Hospitalization 49
12.3 Pregnancy 49
12.3.1 Pregnancy Follow-up 50
12.4 Subject Withdrawal 50
12.5 Reporting Serious Adverse Events 50
13 Statistics **51**
13.1 Methods and Data Analysis 51
13.1.1 Sample Size Justification 51
13.1.2 Study Populations 51
13.1.3 Demographic/Baseline Information 51
13.1.4 Analysis of Safety Data 52
13.1.5 Interim Analysis 52
13.1.6 Pharmacokinetic Analysis 52
13.1.7 Pharmacodynamic Analysis 52
14 Study Management and Data Collection **54**
14.1 Ethical Conduct of the Trial 54
14.2 Institutional Review Board 54
14.3 Subject Informed Consent 54
14.4 Amendments to the Protocol 55
14.5 Study Initiation 55
14.6 Study Monitoring 55
14.7 Case Report Form 56

14.8 Verification Procedures....56
14.9 Retention of Records....57
14.10 Insurance and Indemnity....58
14.11 Audit....58
15 Use of Information....59
15.1 General Aspects....59
15.2 Subject Confidentiality and Data Protection....59
15.3 Final Report and Publication Policy....59
16 References....61

2 LIST OF ABBREVIATIONS AND DEFINITION OF TERMS

Abbreviation	Definition
AE	adverse event
ALT	alanine aminotransferase
AST	aspartate aminotransferase
BMI	body mass index
BP	blood pressure
bpm	beats per minute
CDC	Centers for Disease Control
CL/F	apparent clearance
CLr	renal clearance
C_{min}	minimum plasma concentration
CNS	central nervous system
CRA	clinical research associate
CRO	contract research organization
DCPB	5,11 dihydro 11 chloroacetyl 6H pyrido [2,3 b][1,4]benzodiazepin 6 1
ECG	electrocardiogram
eCRF	electronic case report form
EOS	end of study
ET	early termination
GCP	Good Clinical Practice
GMP	Good Manufacturing Practice
HIV	human immunodeficiency virus
IB	Investigator's Brochure
ICF	informed consent form
ICH	International Council for Harmonisation
IENF	intraepidermal nerve fibers
IENFD	intraepidermal nerve fiber density
IRB	Institutional Review Board
ITT	intent-to-treat
IUD	intrauterine device
IWRS	Interactive Web-Based Response System (IWRS)

K2EDTA	dipotassium ethylenediaminetetraacetic acid
LC/MS-MS	liquid chromatography coupled to tandem mass spectrometry
MedDRA	Medical Dictionary for Regulatory Activities
NF	National Formulary
OCT	optimum cutting temperature compound
PBS	phosphate buffered saline
PGIC	Patient Global Impression of Change
Ph Eur	European Pharmacopoeia
PK	pharmacokinetic
PP	per-protocol
RBC	red blood cell
SAE	serious adverse event
ß-hCG	beta-human chorionic gonadotropin
$t_{1/2}$	half-life
T2DM	type 2 diabetes mellitus
USP	United States Pharmacopoeia
V_z/F	apparent volume of distribution
VAS	Visual Analogue Score
WBC	white blood cell
WHO-DDE	World Health Organization Drug Dictionary Enhanced
λ_z	elimination rate constant

3 INTRODUCTION AND RATIONALE FOR DOSE SELECTION

3.1 Background Information

There are approximately 24 million persons with diabetes in the United States (100 million worldwide), over half of whom will develop some form of nerve dysfunction in their lifetime (CDC, 2007). The most common form of diabetic peripheral neuropathy presents as a distal symmetrical polyneuropathy with a stocking and glove distribution (Tesfaye et al, 2010). Signs and symptoms of diabetic peripheral neuropathy vary depending on the fiber type involved, with large fiber disease impairing proprioception and light touch. Small fiber disease impairs pain and temperature perception, leading to paresthesia, dysesthesias, and/or neuropathic pain. Diabetic peripheral neuropathy may cause muscle weakness and diminished or absent deep-tendon reflexes, especially the Achilles tendon reflex. Advanced diabetic peripheral neuropathy may lead to foot deformities, such as hammer toes, collapse of the mid foot, ulceration, or neuroarthropathy (Charcot joints) (Tesfaye et al, 2010). Ulcerations often go unnoticed for a long period, resulting in infection that spreads to bone, leading to amputation. The immense physical, psychological, and economic costs of diabetic peripheral neuropathy underscore the need for causally targeted therapies (Edwards et al, 2008).

Apart from rigorous control of blood glucose, the only therapies currently approved by Health Canada for the treatment of diabetic peripheral neuropathy are analgesics and antidepressants, which only claim to suppress the pain associated with this disease. These symptomatic treatments do nothing to affect the underlying pathology or the progression of the nerve damage and degeneration associated with the disease itself. There is currently no Health Canada-approved treatment to either prevent diabetes-induced nerve degeneration or promote nerve regeneration. Thus, there is a substantial unmet medical need to develop more effective treatments for diabetic peripheral neuropathy.

WinSanTor, Inc (WinSanTor), has identified the existence of a cholinergic constraint mechanism that suppresses axonal outgrowth and regeneration of sensory neurons. Pirenzepine, a selective M_1 muscarinic receptor antagonist, has been shown to release sensory neurons from this restriction, thereby promoting neurite outgrowth (Calcutt et al, 2017). Pirenzepine is a well characterized muscarinic receptor antagonist, with particular affinity for M_1 receptors (Gastrozepin, 2013). It has limited systemic distribution after oral delivery and does not cross the blood/brain barrier (Tanswell et al, 1984; Hammer and Koss, 1979). The ability of pirenzepine to overcome the endogenous cholinergic constraint on axonal outgrowth to promote nerve regrowth, coupled with a low systemic and central nervous system (CNS) side effect profile, makes it a promising candidate for the treatment of diabetic peripheral neuropathy.

WinSanTor is developing pirenzepine topical solution as a first-in-class therapy for diabetic peripheral neuropathy that not only prevents but reverses the nerve damage caused by type 1 and type 2 diabetes. WinSanTor proposes that topical application of pirenzepine to the calves (mid-calf sock line), ankles and tops and bottoms of feet of subjects with established degenerative diabetic peripheral neuropathy will prevent further progression of the neuropathy and may reverse established neuropathy.

Further summary information on WST-057 is provided in the Investigator's Brochure (IB).

3.2 Rationale for Study and Dose Selection

WinSanTor is developing pirenzepine in a topical formulation as a first-in-class topical therapy for diabetic peripheral neuropathy that not only prevents but heals the nerve damage caused by type 1 and type 2 diabetes. Pirenzepine does not have the major side effect profile expected of an M_1 receptor antagonist because of its relatively poor oral bioavailability and limited blood/brain barrier penetration. Topical application of pirenzepine directly to the site affected by diabetic peripheral neuropathy is intended to maximize local concentration without significantly increasing systemic circulation, thus allowing for efficacy where needed, while minimizing the potential for systemic side effects. We have selected a topical dosage of 2 and 4 mL 4% pirenzepine (73 and 146 mg/day, respectively) based on preclinical efficacy studies and data from an exploratory clinical study with topical oxybutynin, another M_1R antagonist with similar pharmacology.

4 STUDY OBJECTIVES

4.1 Primary Objective

The primary objective of the study is to determine the safety and tolerability of 2 dose levels of WST-057 (4% pirenzepine free base monohydrate solution) after once-daily topical administration in subjects with type 2 diabetes mellitus (T2DM) with peripheral neuropathy.

4.2 Secondary Objectives

The secondary objectives of the study are:

- To evaluate the efficacy of WST-057 to increase intraepidermal nerve fiber density (IENFD) present in punch biopsies collected from the dosing area/treated skin (calf) at weeks 0 and 24 within the same subject.
- To determine the impact (defined as change from baseline) of once-daily topical dosing of WST-057 on quality of life measures using the Norfolk-QOL-DN (Total Score), Norfolk-QOL-DN Physical/Large Fiber Function, Small Fiber Function, Activities of Daily Living, and Symptoms subsets scores at week 24.

4.3 Exploratory Objectives

The exploratory objective evaluated in the study is to determine the impact (defined as change from baseline) of once-daily dosing of WST-057 on quantitative thermal threshold, quantitative vibration perception, visual analogue score (VAS), the change in IENFD in an untreated location (thigh) at week 24 and the Patient's Global Impression of Change (PGIC) at week 24/EOS.

4.4 Pharmacodynamic Endpoints

The following pharmacodynamic endpoints will be assessed for change from baseline:

- IENFD collected at baseline and week 24 collected from the dosing area (calf) and the nondosing area (thigh)
- Quality of life measurement (Norfolk-QOL-DN) Total Score
- Norfolk-QOL-DN Physical/Large Fiber Function subset
- Norfolk-QOL-DN Small Fiber Function subset
- Norfolk-QOL-DN Activities of Daily Living subset
- Norfolk-QOL-DN Symptoms subset
- Toronto Clinical Neuropathy Score
- Modified Toronto Clinical Neuropathy Score
- Visual Analogue Score
- Quantitative thermal threshold
- Quantitative vibration perception

- PGIC

4.5 Safety Endpoints

The primary safety endpoints of this study are:

- Adverse events (AE)
- Dermal Draize Score
- Clinical laboratory tests (serum chemistry, hematology, and urinalysis)
- Vital signs
- Body weight
- 12-lead electrocardiogram (ECG)
- Physical examination
- Concomitant medications

5 STUDY DESCRIPTION

5.1 Study Design

This is a randomized outpatient, double-blind, placebo-controlled, multiple-site study of the safety, tolerability, and exploratory efficacy of topically administered WST-057 (4% pirenzepine free base monohydrate) for 24 weeks in subjects with T2DM with peripheral neuropathy. Subjects will attend visits at screening (day -45 to -28); day 1 (baseline); weeks 2, 4, 8, 12, 16, 20, 24; and follow-up (week 26). Approximately 60 subjects with T2DM with peripheral neuropathy will be randomized to 1 of 4 treatment groups in a 1:1:4:4 ratio: placebo control 2 mL (n = 6 subjects), placebo control 4 mL (n = 6 subjects); low dose (2 mL) WST-057 (73 mg pirenzepine free base monohydrate) (n = 24 subjects); and high dose (4 mL) WST-057 (146 mg pirenzepine free base monohydrate) (n = 24 subjects), with the assumption that a total of 50 subjects will complete the study.

This study is designed with 4 periods: screening, baseline/day 1, outpatient treatment, and safety follow-up.

Screening Period (Days -45 to -28)

Subjects will be screened for eligibility during a screening period that must be completed 28 days before baseline/day 1 (ie, the screening biopsy must be taken no later than day -28 in order to allow the screening biopsy to heal). During the screening period, all necessary assessments will be performed and subject eligibility for entry into the double-blind portion of the study will be verified. Subjects who do not qualify will be discontinued and considered screen failures. If during screening, a subject fails to meet any inclusion or exclusion criteria, then the Investigator is not required to complete the remaining screening activities. The following procedures will be performed during the screening period:

- Obtain signed informed consent from the subject before any study-specific procedures are performed
- Medical history review with the subject
- Demographic information collection
- Inclusion and exclusion criteria review
- Toronto Clinical Neuropathy Score
- Modified Toronto Clinical Neuropathy Score
- Sural nerve amplitude (accepted if > 1 μV)
- Skin Biopsy for IENFD (accepted if > 1 and < 10 IENF/mm)
- Quality of Life (Norfolk-QOL-DN)
- Quantitative thermal threshold (screening)
- Quantitative vibration perception (screening)

- Dermal Draize Score (screening)
- Prior and concomitant medications review
- Complete physical examination, including height and body weight (for body mass index [BMI] calculation)
- Supine and standing vital signs
- 12-lead ECG
- Serum pregnancy test (women of childbearing potential only)
- Clinical laboratory tests
- Serology for hepatitis B surface antigen, hepatitis C antibody, and human immunodeficiency virus (HIV) 1 and 2 antibody
- Urine drug screen and alcohol breathalyzer test
- AEs
- Verify that subject is physically able to inspect lower leg, upper and lower foot for wounds, infections, or other anomalies, and will be able to self-administer the investigational drug

Patient Check-in (Baseline/Day 1)

If during the Baseline visit, a subject fails to meet any inclusion or exclusion criteria, then the Investigator is not required to complete the remaining visit activities. On the morning of day 1 (predose), the following procedures will be performed:

- Review of IENFD results from the screening calf biopsy (accepted if > 1 and < 10 IENF/mm)
- Verify baseline biopsy site has healed (prior to dosing phase)
- Medical history review
- Inclusion/exclusion review
- Urine drug screen and alcohol breathalyzer
- Urine dipstick pregnancy test
- Concomitant medications and AEs
- Physical examination and body weight, height, BMI calculation
- Supine and standing vital signs
- Toronto Clinical Neuropathy Score
- Modified Toronto Clinical Neuropathy Score
- 12-lead ECG
- Clinical laboratory tests (hematology, clinical chemistry, urinalysis)

- Serology for hepatitis B surface antigen, hepatitis C antibody, HIV 1 & 2 antibody
- Quantitative thermal threshold
- Quantitative vibration perception
- Visual Analogue Score
- Sural nerve testing
- Quality of Life (Norfolk-QOL-DN)
- Dermal Draize Score (baseline/day 1)
- Randomization and receipt of assigned bottle of investigative product
- Instruct subjects on the proper storage and self-administration of study drug
- Patient training on the topical application of investigational drug

Dosing Period (Day 2 to Week 24 ± 3 days)

Throughout the dosing period, safety assessments will be performed and plasma samples will be collected to determine the trough concentrations of WST-057. The acceptable visit window is ± 3 days. The following additional assessments and procedures will be completed:

- Review dosing procedure (weeks 2, 4, 8, 12, 16, 20, and 24)
- Study drug dosing (day 1 to week 24)
- Physical examination and body weight, BMI calculation (weeks 2, 4, 8, 12, 16, 20, and 24)
- Vital signs (weeks 2, 4, 8, 12, 16, 20, and 24)
- 12-lead ECG (weeks 2, 4, 8, 12, 16, 20, and 24)
- Clinical laboratory tests (weeks 2, 4, 8, 12, 16, 20, and 24)
- Trough plasma sample for WST-057 pharmacokinetic (PK) analysis (weeks 2, 4, 8, 12, 16, 20, and 24)
- Quality of life (Norfolk-QOL-DN) (weeks 2, 4, 8, 12, 16, 20, and 24)
- Toronto Clinical Neuropathy Score (weeks 2, 4, 8, 12, 16, 20, and 24)
- Modified Toronto Clinical Neuropathy Score (weeks 2, 4, 8, 12, 16, 20, and 24)
- Visual Analogue Score (weekly 1-24)
- Dermal Draize Score (weeks 2, 4, 8, 12, 16, 20, and 24)
- Review of concomitant medications and AEs (weeks 2, 4, 8, 12, 16, 20, and 24)
- Dispense new bottle of assigned investigative product (weeks 4, 8, 12, 16, and 20)
- Subject is to return the remaining unused investigational drug or empty container to the Investigator (weeks 4, 8, 12, 16, 20, and 24)

End-of-study Procedures (Early Termination or Week 24 ± 3 days)

- Physical examination and body weight, BMI calculation (week 24)
- Vital signs (week 24)
- 12-lead ECG (week 24)
- Clinical laboratory tests (week 24)
- Trough plasma sample for WST-057 PK analysis (week 24)
- Serum pregnancy test (women of childbearing potential) (week 24)
- Concomitant medications review (week 24)
- Adverse events review (week 24)
- Dermal Draize Score (week 24)
- Visual Analogue Score (week 24)
- Toronto Clinical Neuropathy Score (week 24)
- Modified Toronto Clinical Neuropathy Score (week 24)
- Sural nerve amplitude (week 24)
- Skin Biopsy for IENFD (week 24)
- Quality of Life (Norfolk-QOL-DN) (week 24)
- Quantitative thermal threshold (week 24)
- Quantitative vibration perception (week 24)
- Patients' Global Impression of Change (week 24)

Safety Follow-up (+ 10 days ± 3 days)

Subjects who complete treatment will have a safety follow-up visit performed on week 26, during which the following assessments will be performed:

- Symptom-directed physical exam
- Concomitant medications and AEs
- Biopsy examination and suture removal, if necessary

In addition, all subjects, including those who prematurely discontinue study drug, will be contacted via telephone 28 (± 3) days after the last dose of study drug.

5.2 Stopping Criteria

Dose administration will be discontinued immediately according to the following criteria.

Individual Stopping Criteria

1. Any subject with an AE that is unexpected and/or may pose a significant safety risk, including abnormal ECG and/or drug-induced liver injury, or any other event considered by the Investigator to pose a significant risk to the subject.
2. Presence of a serious AE (SAE) considered by the Investigator to be related to the investigational product.

Overall Study Stopping Criteria

1. Three or more subjects develop the same SAE considered by the Investigator to be related to the investigational product.
2. Any clinical observation that the Investigator considers a safety issue such that continued study drug administration comprises a medically significant or unacceptable risk for the study participant(s).

5.3 Schedule of Assessments

The time and events schedule for this study is provided in Table 1.

Table 1. Schedule of Time and Events for the Study*

	Screening	Baseline			Outpatient Phase					EOS & ET[a]	Follow-up
**Study Day**	**Day -45 to - 28**	**1**	**Study Week**	**2**	**4**	**8**	**12**	**16**	**20**	**24**	**26[m]**
Event/assessment											
Informed consent	X										
Medical history and demographics	X	X									
Inclusion/exclusion criteria	X	X									
Prior/concomitant medications	X	X		X	X	X	X	X	X	X	X
Physical examination	X	X		X	X	X	X	X	X	X	X[b]
Body weight, height, and BMI calculation	X	X[c]		X[c]	X[c]	X[c]	X[c]	X[c]	X[c]	X[c]	
Vital signs measurements	X	X		X	X	X	X	X	X	X	
12-lead ECG	X	X		X	X	X	X	X	X	X	
Clinical laboratory tests	X[d]	X[d]		X[d]	X[d]	X[d]	X[d]	X[d]	X[d]	X[d]	
Serology for hepatitis B surface antigen, hepatitis C antibody, HIV 1 and 2 antibody	X	X									
Pregnancy test (women of childbearing potential only)	X[e]	X[e]								X[e]	
Urine drug screen and alcohol breathalyzer test	X	X									
Baseline biopsies	X[f,g]										
Sural nerve testing	X	X								X	
Quantitative thermal threshold	X	X								X	
Quantitative vibration threshold	X	X								X	
Toronto Clinical Neuropathy Score	X	X		X	X	X	X	X	X	X	
Modified Toronto Clinical Neuropathy Score	X	X		X	X	X	X	X	X	X	
Quality of life (Norfolk-QOL-DN)	X	X		X	X	X	X	X	X	X	
Check-in		X									
Randomization		X[h]									
Study drug administered (day 1 to week 24)		X[i]		X[i]	X[i]	X[i]	X[i]	X[i]	X[i]	X[i]	

Table 1. Schedule of Time and Events for the Study*

	Screening	Baseline			Outpatient Phase					EOS & ET[a]	Follow-up
**Study Day**	**Day -45 to - 28**	**1**	**Study Week**	**2**	**4**	**8**	**12**	**16**	**20**	**24**	**26[m]**
Adverse event monitoring	X	X		X	X	X	X	X	X	X	X
Dermal Draize Score	X[j]	X[j]		X[j]	X[j]	X[j]	X[j]	X[j]	X[j]	X[j]	
Pharmacokinetic sampling (WST-057)				X[k]	X[k]	X[k]	X[k]	X[k]	X[k]	X[k]	
EOS biopsies[l]										X[l]	
Patients' Global Impression of Change (PGIC)										X	
Visual Analogue Score		X[n]		X[n]	X[n]	X[n]	X[n]	X[n]	X[n]	X[n]	

BMI = body mass index; ECG = electrocardiogram; EOS = end of study; ET = early termination; HIV = human immunodeficiency virus

* = It is acceptable to change the schedule of assessments in order to accommodate local and global health concerns and ensure patient safety due to Covid19.
[a] = The ET and EOS visits (week 24) will follow the same procedures.
[b] = Symptom directed physical examination.
[c] = Body weight and BMI calculation only. Height is taken at screening only.
[d] = Blood samples will be collected for serum chemistry and hematology, and urine will be collected for urinalysis (refer to section 7.2.2) at each visit. Subjects will be required to fast for at least 12 hours prior to collection.
[e] = For women of childbearing potential, a serum pregnancy test will be performed at screening and EOS/week 24. A urine pregnancy test will be performed at the Baseline visit.
[f] = Two standard 3-mm skin biopsies will be collected during the Screening visit from the distal leg or calf; approximately 10 cm above the malleolus and 3 cm to the posterior; and from the untreated area on the lateral thigh, approximately 25 cm below the iliac spine, approximately 5 cm below the level of the pubis and 8 cm to the posterior.
[g] = Prophylactic antibiotics may be administered to participants who, in the judgment of the Investigator, are considered to be at high risk for infection of the biopsy site.
[h] = Randomization will be prior to dose administration on day 1.
[i] = Investigational product will be administered by the subject by dispensing material from pump into palms of the hand and then spreading evenly over the calves (mid-calf sock-line), ankles and tops, and bottoms of feet.
[j] = Skin irritation (including dryness, redness, swelling, itching, burning/stinging) at the application site will be assessed using a Dermal Draize Scale prior to dosing on visit days.
[k] = Trough blood samples (1 sample at each specified visit) will be collected for pharmacokinetic analysis.
[l] = Two standard 3-mm skin biopsies will be collected during the EOS visit for early terminations or the week 24 visit. The biopsies will be collected at a distance of no less than 2 cm away and no further away than 3.5 cm from the baseline biopsy location.
[m] = The biopsy sites will be examined at the 26-week visit to monitor healing and remove sutures, if necessary. If the sutures are not suitable for removal, another visit for suture removal will be scheduled.
[n] = The visual analogue score will be collected weekly via a mobile phone application completed by the patient at the same time each week.

6 SUBJECT ELIGIBILITY AND WITHDRAWAL CRITERIA

6.1 Number of Subjects

Approximately 60 subjects with T2DM with peripheral neuropathy will be randomized to 1 of 4 treatment groups in a 1:1:4:4 ratio: placebo control 2 mL (n = 6 subjects); placebo control (4 mL) (n = 6 subjects); low dose WST-057 (73 mg pirenzepine free base monohydrate) (n = 24 subjects); and high dose WST-057 (146 mg pirenzepine free base monohydrate) (n = 24 subjects), with the assumption that a total of 50 subjects will complete the study.

6.2 Inclusion Criteria

Subjects must meet all of the following inclusion criteria to be eligible to participate in the study:

1. Diagnosis of T2DM (as defined by the 2013 Diabetes Canada guidelines).
2. Male and female patients in the age range of 18 to 75 years (inclusive).
3. Presence of definite diabetic neuropathy (as defined by the Toronto Consensus Guidelines) of at least 12 months duration in the lower extremities.
4. Provide written informed consent prior to entering the study or undergoing any study procedures.
5. Females should be either not of childbearing potential as a result of surgery or menopause (1 year after onset), or of childbearing potential and must be practicing a highly effective medically acceptable method of contraception (e.g. abstinence, or hormonal contraceptives (e.g, combined oral contraceptives, patch, vaginal ring, injectables, and implants); intrauterine device (IUD) or intrauterine system (IUS); or vasectomy (partner)) for at least 1 month before the screening visit and for 1 month after the last dose of study drug. If access or use of a highly effective medically acceptable method of contraception is not achievable, then a combination of barrier methods (e.g. male condom, female condom, cervical cap, diaphragm, contraceptive sponge) is acceptable (e.g. male condom with diaphragm, male condom with cervical cap). Eligible female subjects must also have a negative serum beta-human chorionic gonadotropin (ß-hCG) at the screening visit.
6. Males must use an acceptable form of contraception (e.g. male condom with diaphragm, male condom with cervical cap, or male condom in association with spermicide)
7. Glycemic control has been optimized and has been stable for at least three months prior to randomization. Optimal glycemic control refers to the best possible diabetic control that an individual patient can attain with usual standards of care, which usually takes more than two months to establish.
8. Patients must have a screening IENF density range of no less than 1 IENF/mm and no more than 10 IENF/mm.
9. Participating subjects must be reliable, willing, and able to cooperate with all study procedures, including the following:
    - Return for study visits on the required dates

- Be physically able to inspect calves, tops of ankles, and soles of feet for wounds, infections, or other anomalies, and be able to self-administer the investigational drug to calves and feet.
- Be able to accurately and reliably report symptoms (including treatment-emergent signs and symptoms).
- Take study drug as required by protocol.

10. Be on stable antidiabetic treatment (insulin, oral agents, or lifestyle) that is not anticipated to change during the course of the study, except if medically required.
11. Be on stable analgesic treatment (same medication and dose) or stable nonpharmacological pain treatment for at least 4 weeks prior to screening and remain on this stable treatment throughout the study (unless otherwise directed by a physician). Nonpharmacologic pain treatment includes the following: relaxation/hypnosis, physical or occupational therapy, counseling, etc. Episodic or periodic treatments, such as monthly injections for treatment of pain (eg, local anesthetics), will not be permitted. Topical anesthetics/analgesics such as capsaicin, topical cannabinoid (CBD) oil or extracts, lidocaine patches and compounded topical applications are also not allowed.
12. General health status must be acceptable for participation in this 24-week clinical study, with no hospitalizations for medical conditions within 12 weeks before and during screening per judgment of the Investigator. Any question regarding eligibility will be addressed with the medical monitor.
13. Fluency (oral and written) in the language in which the standardized tests will be administered.

6.3 Exclusion Criteria

Subjects who meet any of the following exclusion criteria will be excluded from participating in the study:

1. Lower leg IENFD at screening of < 1 or >10 IENF/mm.
2. Uncontrolled glycemia
3. Proliferative retinopathy or maculopathy requiring acute treatment.
4. Requiring dialysis.
5. Impaired liver function, defined as aspartate aminotransferase (AST) or alanine aminotransferase (ALT) ≥ 3 times the upper limit of normal.
6. Presence of clinically significant peripheral or autonomic neuropathy that is clearly of nondiabetic origin.
7. Uncontrolled treated/untreated hypertension (systolic blood pressure [BP] ≥ 180 or diastolic BP ≥ 100 at screening).
8. Amputations of lower extremities or presence of foot ulcers.
9. Clinically significant active macrovascular disease, including myocardial infarction or cerebrovascular event within the past 12 months.

10. Uncontrolled or untreated hypothyroidism.
11. Active infection (eg, HIV, hepatitis), or a history of severe infection during the 30 days prior to screening.
12. Evidence of severely immunocompromised status.
13. Major surgical procedure during the 90 days prior to screening.
14. Diagnosis and/or treatment of malignancy (except for basal cell or squamous cell skin cancer, in-situ carcinoma of the cervix, or in-situ prostate cancer) within the past 5 years.
15. Clinically significant gastric emptying abnormality (eg, severe gastroparesis).
16. Urinary retention or an enlarged prostate.
17. Uncontrolled glaucoma.
18. Other clinically significant, active (over the past 12 months) disease of the gastrointestinal, pulmonary, neurological, genitourinary, endocrine, rheumatologic or hematological system that, in the opinion of the Investigator, would compromise the subject's participation in the study, might confound the results of the study, or pose additional risk in administering the study drug.
19. New treatment with (< 3 months) antioxidant supplements, vitamins, or drugs known to affect oxidative stress and peripheral diabetic neuropathy such as Superoxide Dismutase, Alpha Lipoic Acid, Acetyl L-Carnitine and Vitamin B12.
20. Known or suspected history of alcohol or substance abuse. Moderate and stable consumption of alcohol, cannabis and nicotine are not exclusionary
21. Mental incapacity, unwillingness, or language barrier precluding adequate understanding of or cooperation with the study.
22. Women of childbearing potential who are pregnant, breast-feeding, or intend to become pregnant. Women of childbearing potential must have a negative pregnancy test at Screening and must agree to use adequate contraceptive methods during the study and for 1 month after the last dose of study drug (see inclusion criterion 5).
23. History of allergy or sensitivity to M_1 antagonists or anticholinergics in general or any of the components of the investigational product formulations.
24. Known allergy or hypersensitivity to pirenzepine or another component of the investigational product.
25. History of sensitive skin, as defined by a requirement to use soap and skin products formulated for "sensitive skin".
26. Currently taking any medicines to treat overactive bladder (anticholinergic agents, such as Gelnique).
27. Failure or inability to perform screening or baseline assessments.
28. Patients with any condition that could potentially interfere with the conduct of the study or confound efficacy evaluations, including the following as specified in numbers 29 through 35 below:

29. Pain or neuropathy from another cause (as determined by the investigator) (including central pain, radiculopathy, painful arthritis, autoimmune and inflammatory diseases including rheumatoid arthritis, lupus, Sjogren's syndrome, vasculitic disorders such as periarteritis nodosa, Churg-Strauss, etc., celiac disease, Crohn's disease, ulcerative colitis, spondyloarthropathies, sarcoidosis, etc).
30. Skin or soft-tissue lesions in the area affected by neuropathy that are painful or could alter sensation.
31. Systemic infections (eg, HIV, hepatitis, tuberculosis, syphilis).
32. Exposure to an experimental drug, experimental biologic, or experimental medical device within 3 months before screening.
33. Any open wound(s) and/or sunburn(s) in the dosing area. Subjects who have a wound and/or sunburn at screening that is anticipated to resolve before day -1 can be enrolled.
34. History of a serious skin disease (as determined by the Investigator), such as skin cancer, psoriasis, eczema or stasis dermatitis.
35. Receipt of a tattoo in the dosing area within 12 months of dosing.

6.4 Withdrawal Criteria

The subject has the right to withdraw from the study at any time.

The subject will be withdrawn from the study if 1 or more of the following occurs:

1. A deterioration in the subject's signs/symptoms and/or the subject develops a disease or condition that, in the opinion of the Investigator, would compromise the subject's safety if he/she continued in the study.
2. In the Investigator's judgment, it is in the best interest of the subject.
3. Violation of the protocol inclusion and/or exclusion criteria as deemed relevant by the Investigator and discussed with the medical monitor.
4. The subject begins to take any medication(s) that is excluded by the protocol.
5. Any of the conditions are met in section 5.2, Stopping Criteria, in the judgement of the Investigator.
6. Sponsor discontinues the study.

If a subject withdraws prematurely from the trial due to the above criteria or any other reason, study staff should make every effort (at withdrawal) to complete the full set of evaluations scheduled for the final day of the treatment period. The reason for subject withdrawal must be documented in the electronic case report form (eCRF).

If a subject withdraws from the study due to an AE (eg, clinical signs, symptoms, or clinically significant laboratory abnormality), the subject will be asked, at a minimum, to return to the clinic for the evaluations scheduled for the post-treatment follow-up period. If the AE has still not resolved, additional follow-up will be performed, as appropriate, and documented in the

subject’s medical records. As a minimum requirement, AEs should be followed for 30 days after the subject’s last dose of study medication.

In the case of a subject lost to follow-up, attempts to contact the subject must be made and documented in the subject’s medical records.

Withdrawn subjects will be replaced as needed to meet enrollment expectations.

7 STUDY PROCEDURES AND ASSESSMENTS

7.1 Pharmacodynamic and Pharmacokinetic Evaluations

The pharmacodynamics of WST-057 will be assessed by evaluating the change from baseline in the following parameters:

- IENFD change from baseline for both treated and untreated skin (section 7.1.1)
- Quantitative thermal threshold (section 7.1.2)
- Quantitative vibration perception (section 7.1.2)
- Patients' Global Impression of Change (section 7.1.2)
- QOL measurement (Norfolk-QOL-DN) and subset analysis (section 7.1.3)
- Toronto Clinical Neuropathy Score (section 7.1.3)
- Modified Toronto Clinical Neuropathy Score (section 7.1.3)
- Visual Analogue Score (section 7.1.3)

7.1.1 Intraepidermal Nerve Fiber Density

Change in IENFD from baseline to week 24 within each subject will be evaluated via a skin punch biopsy. The baseline punch biopsy will be collected at least 28 days prior to study day 1, such that the biopsy healing can be monitored and confirmed prior to first dose administration.

The skin punch biopsies should be performed in the calf approximately 10 cm above the lateral malleolus and 3 cm to the posterior; and in the thigh approximately 25 cm below the iliac spine, 5 cm below the level of the pubis, and 8 cm to the posterior, using a 3 mm disposable punch under sterile technique after topical anesthesia with lidocaine. The biopsy should be shallow (3 to 4 mm), and should require no suture (Lauria et al, 2010).

If possible, the end-of-study biopsy should be collected no less than 2 cm and no more than 3.5 cm from the initial biopsy site from the same leg as the screening biopsies. Additional biopsy collection details will be outlined in a study procedure document provided to the site.

Upon collection, the biopsy should be immediately fixed in cold Zamboni's fixative solution and stored overnight in a 4°C refrigerator. The biopsy should remain in this chilled fixative for a minimum of 12 hours and a maximum of 24 hours. Next, the fixed skin sample should be transferred to chilled cryovial containing 1 × phosphate buffered saline (PBS) with 15% sucrose. Additional processing details will be outlined in a study procedure document provided to the site.

The sample will then be shipped with a wet ice pack in an insulated container for central processing to the following address:

WinSanTor, Inc
Attn: Katie Frizzi (UCSD)
3830 Valley Centre Dr. #705-632

San Diego, CA 92130
United States of America
Telephone: +1.858.822.5797

Upon receipt at the laboratory, the samples will be transferred to a labeled cryovial containing 1 × PBS with 30% sucrose and stored in a 4°C refrigerator. Following completion of the sucrose protectant phase, the biopsy sample will be embedded in a 15 mm × 15 mm standard cryo-mold filled with optimum cutting temperature compound (OCT) and frozen. These blocks will be stored in a -80°C freezer until cutting. The tissue will be cut and stained according to the methods and using the materials outlined in Beiswenger et al, 2008.

Nerves present in the stained tissue will be counted by a qualified, double-blinded observer. The IENFD will be counted according to the accepted clinical counting methodology; counting only fibers originating in the dermis and crossing the dermis/epidermis junction into the epidermis (Lauria et al, 1999). The methods for biopsy and slide processing and reading will be outlined in a study procedures document outside of this protocol.

The length of each sample will be measured in mm in order to determine IENF/mm. Additional sample receipt, processing, and reading details will be outlined in a study procedure document provided to the site.

7.1.2 Quantitative Sensory Testing

The change from baseline in quantitative cooling detection threshold and vibration perception threshold will be evaluated according to the Schedule of Assessments. The method of limits will be used.

Quantitative Thermal Threshold

Cooling detection thresholds will be measured using the TSA-II Neurosensory Analyser (Medoc Advanced Medical Systems, Ramat-Yishai, Israel), or comparable device, and the method of limits. A stimulator with a baseline temperature of 32°C will be applied to both feet. The temperature will be gradually decreased until the subject detects a cooler temperature—this constitutes the cooling threshold. Cooling thresholds are reliable and sensitive to detect small fibre dysfunction. The average of the 3 levels, will be calculated and reported for both feet.

Quantitative Vibration Perception

Vibration perception threshold testing, (a form of quantitative sensory testing for large nerve fibres) will be done using the Neurothesiometer (Horwell Scientific, London, UK), or comparable device, and applying the method of limits. A trained technician will place a stimulation probe on the pulp of the first toe of each foot, progressively increasing the vibration intensity until the subject perceives it. The examiner must ensure that the subject is able to distinguish vibration from applied pressure. The technician will record 3 measurements at each stimulation site and then report the average for both feet.

Sural Nerve Testing

Sural nerve testing will be assessed according to the Schedule of Assessments. Sural nerve testing measures the sural nerve conduction velocity and sensory nerve action potential, which have been shown to be sensitive indicators of nerve degeneration in patients with diabetes. Low sural nerve conduction velocity and sensory nerve action potential are associated with diabetic peripheral neuropathy (Poulose et al, 2015).

Sural nerve conduction and velocity studies will be conducted according to manufacturers' instructions for the respective device. A sural nerve conduction velocity in m/s and a sural nerve amplitude in µV will be recorded for each leg. If necessary, per the instructions or requirements of the device, a series of 3 responses will be collected, and the average reported for each measure for each leg.

Patients' Global Impression of Change

The Patients' Global Impression of Change (PGIC) scale will be used to assess patients' perception of changes following treatment (i.e., "feeling better" or "feeling worse"). The PGIC is a 7-point verbal scale. The PGIC is commonly used in clinical studies assessing pain relief following treatment, due to its ease of administration and scoring and because it is a generic scale applicable to a wide variety of conditions and treatments including diabetic peripheral neuropathy (Perrot and Lanteri-Minet, 2018) It has been demonstrated that the subjective "much improved" and "very much improved" ratings indicate moderately important and substantial improvement (Dworkin et al, 2008).

In this study, the PGIC will be administered to the patients on paper by site personnel at Study Week 24/EOS

7.1.3 Disease Assessment Scales

The change from baseline in the Norfolk-QOL-DN will be assessed according to the Schedule of Assessments. The Norfolk-QOL-DN measures a subject's perceptions of the effects of diabetic neuropathy.

The Toronto Clinical Neuropathy Score and Modified Toronto Clinical Neuropathy Score will be evaluated according to the Schedule of Assessments.

7.1.4 Pain Assessment Scale: Visual Analogue Score

The visual analog scale (VAS) is a pain rating scale first used by Hayes and Patterson in 1921. Scores are based on self-reported measures of symptoms that are recorded with a single mark placed at one point along the length of a line that represents a continuum between the two ends of the scale—"no pain" on the left end of the scale and the "worst pain" on the right end of the scale. Measurements from the starting point (left end) of the scale to the patients' marks are recorded and are interpreted as their level pain. The self-reported values can be used to track pain progression for a patient or to compare pain between patients with similar conditions.

In this study, the VAS will be administered to the patients via a mobile device application. The application will be password protected or biometrically locked such that only the subject is able to input his or her data. The device will prompt the subject to input his or her level of pain at the screening visit and on a weekly basis thereafter until week 24 or the early termination visit.

7.2 Safety Evaluations

Safety and tolerability of WST-057 will be assessed for the primary objective using the following assessments:

- AEs (section 7.2.1)
- Clinical laboratory tests (section 7.2.2)
- Vital signs (section 7.2.3)
- Physical examination (section 7.2.4)
- Body weight, height, and BMI (section 7.2.7)
- 12-lead ECG (section 7.2.3)
- Concomitant medications (section 10)
- Dermal Draize Score (section 7.2.5)

7.2.1 Adverse Events

The Investigator will determine during the course of all study periods whether any AEs have occurred. The subjects will be questioned in a general way and no specific symptoms will be suggested. Slight flushing and/or a tingling or other altered sensations in the dosing area is expected and is not considered an AE. Section 11 contains additional information with regard to AEs. AEs will be captured from the signing of the informed consent form (ICF).

7.2.2 Clinical Laboratory Tests

Clinical laboratory tests will be performed by a qualified laboratory. Blood and urine samples will be taken prior to the dosing (on dosing days) at the clinic visits indicated for clinical chemistry, hematological analysis, and urinalysis. Subjects should be fasted prior to the collection of samples (12 hours prior to sample collection).

The following parameters will be measured:

Hematology:	Hematocrit, hemoglobin, red blood cell (RBC) count, white blood cell (WBC) count, differentials (neutrophils, eosinophils, basophils, lymphocytes, and monocytes), platelet count, and reticulocytes
Clinical Chemistry: (Chem 20)	Albumin, alkaline phosphatase, ALT, AST, blood urea nitrogen, calcium, chloride, CO_2, creatinine, direct bilirubin, gamma-glutamyl transferase, glucose, lactate dehydrogenase, phosphorus, potassium, sodium, total bilirubin, total cholesterol (triglycerides and HDL/LDL cholesterol including total cholesterol), total protein, uric acid
Urinalysis:	Color, specific gravity, pH, glucose, ketones, protein, bilirubin, urobilinogen, WBCs, RBCs, and microscopy

For females of childbearing potential, a serum pregnancy test will be performed at screening and at the end of study/week 24 visit, and a urine pregnancy test at baseline.

Serology for hepatitis B surface antigen, hepatitis C antibody, and HIV 1 and 2 antibody will be conducted at screening and baseline to confirm eligibility.

A urine drug screen will be performed at screening and baseline. Urine samples will be collected in a container labeled “URINE DRUG SCREEN”.

Study site SOPs for requesting and documenting clinical laboratory samples will be followed for this study.

Laboratory reports should be reviewed, signed, and dated by the Investigator or delegated physician. Each abnormal result will be assessed as clinically or not-clinically significant and a comment will be provided for all out-of-range analytes deemed clinically significant (ie, document probable cause for abnormal results). Laboratory data will be collected electronically and transferred to the contract research organization (CRO) handling the case report form data for integration with the eCRF data.

7.2.3 ECG and Vital Signs

A 12-lead ECG will be obtained at the visits indicated in Schedule of Assessments and will include ventricular rate (beats per minute [bpm]), PR, QRS, QT, QTc, QTcF, and QTcB interval measurements and calculations.

The 12-lead ECGs will be digitally recorded after 10 minutes rest in the sitting position. All ECGs will be over-read by an appropriately qualified clinical staff physician and the data entered in the subject’s CRF or via validated direct electronic transfer for integration with the eCRF data.

ECG findings will be classified as normal or abnormal and associated interpretive remarks will be captured. The clinical significance of ECG findings will be determined by the Investigator after review of the ECG report with relation to the subject’s medical history, physical examination, and concomitant medications.

Vital signs (BP [mmHg], respiratory rate [breaths per minute], temperature [°F], and heart rate [bpm]) will be measured at the clinic visits or time points indicated in the Schedule of Assessments. Blood pressure will be measured after at least a 5-minute rest in the sitting position. Blood pressure will be measured with a standard mercury sphygmomanometer or an automated oscillometric BP monitor.

7.2.4 Physical Examination

A physical examination will be performed at the time points indicated in the Schedule of Assessments. This evaluation will include examination of the following body systems: general appearance; eyes, ears, nose, and throat; head and neck; chest and lungs; cardiovascular; abdomen; musculoskeletal; lymphatic (head and neck); dermatological (including the palms of hands to evaluate any investigational product reaction); neurological; and extremities. Urogenital and psychiatric abnormalities will be accessed as part of the subject's medical history at screening, and any abnormalities (ie, if the subject has a complaint for that system) in these systems during the treatment period will be recorded as AEs.

The physical examination at the follow-up visit (week 26) will be a symptom directed examination.

7.2.5 Local Skin Irritation Assessment (Dermal Draize Score)

No local skin irritation is expected to be observed based on nonclinical studies of primary dermal toxicity and a dermal sensitization. However, as noted in the physician's insert for Gastrozepin (50 mg oral pirenzepine), a flushing or slight redness may occur at the test site.

The application site will be monitored for signs of irritation, including dryness, redness, swelling, and itching/burning. The Dermal Draize Scale will be used for recording of skin reactions (Appendix A).

7.2.6 Demographics and Medical History

Demographics and brief, relevant medical and social history will be taken at screening, including tobacco use and alcohol consumption.

7.2.7 Body Weight, Height, and Body Mass Index

Body weight and height (screening only) will be measured at the time points indicated in the Schedule of Assessments. BMI will be calculated and used to confirm eligibility using body weight from the screening visit and height from the screening visit, using the following formula:

$$\frac{weight(kg)}{height^2(m^2)} \quad \text{OR} \quad \frac{weight(pounds) \times 703}{height^2(inch^2)}$$

7.3 Pharmacokinetic Evaluations

Trough blood samples will be collected at the time points indicated in the Schedule of Assessments. The concentration of pirenzepine in these samples will be analyzed by a contract bioanalytical laboratory using a validated liquid chromatography coupled to tandem mass spectrometry (LC/MS-MS) method. Full details of the methodology will be presented in the bioanalytical report, which will be appended to the clinical study report. The data obtained from these analyses may be used to calculate the following pharmacokinetic parameters: minimum plasma concentration (C_{min}), elimination rate constant (λ_z), half-life ($t_{1/2}$), apparent clearance (CL/F), and apparent volume of distribution (V_z/F).

7.4 Data Monitoring Committee

A formal data monitoring committee is not planned for this study.

7.5 Total Blood Volume

The total volume of blood obtained from an individual study participant is expected to be approximately 105 mL, including PK samples (14 mL) and clinical laboratory tests (91 mL).

7.6 Pharmacokinetic Sampling

Samples of venous blood will be obtained in dipotassium ethylenediaminetetraacetic acid (K2EDTA) tubes (a minimum of 2 mL per time point required) at the time points indicated in the Schedule of Assessments.

Immediately after collection, the tube will be gently inverted several times to mix the anticoagulant with the blood sample. The plasma fraction will be separated by placing the collection tube into a refrigerated centrifuge (4°C to 8°C) for 10 minutes at approximately 1500 × g. The plasma fraction will be withdrawn by pipette and divided into 2 polypropylene freezing tubes (designated primary and secondary samples), with each tube receiving approximately equal aliquots. All sample collection and freezing tubes will be clearly labeled in a fashion that identifies the subject, the study period, and the collection time. Labels will be fixed to freezing tubes in a manner that will prevent the label from detaching after freezing. All plasma samples will be processed and placed into a freezer (< -20°C) within 1 hour after collection.

All plasma samples will be stored frozen (< -20°C) until they are shipped to the analytical facility. Prior to shipping, the samples will be packed into thermal insulated containers and packed in sufficient dry ice to assure that they remain frozen for a minimum of 72 hours and protected from breakage during shipment, and will be accompanied by an Excel spreadsheet manifest detailing the contents of each shipment. The bioanalytical facility will be notified of the shipment via e-mail or facsimile. Samples will be shipped by overnight priority courier. The samples will be divided into 2 shipments, each containing 1 aliquot of plasma for each time point. After receipt of verification that the first shipment was received by the analytical facility, the second shipment will be sent. On the day of shipment, the site will e-mail notice to the laboratory and sponsor with the tracking number and a copy of the shipping manifest.

All samples will be shipped to:

Q2 Solutions
Attn: Valerie L Belcher
19 Brown Road
Ithaca, NY 14850
USA
valerie.belcher@q2labsolutions.com
Office: +1 607 442 4544
Mobile: +1 512 840 9961

8 CONDUCT OF STUDY

8.1 Study Blinding and Randomization

Subjects who fulfill the eligibility criteria and provide informed consent will be enrolled into the study and randomized in a double-blind manner at the study site. Subjects will be randomized to placebo control 2 mL; placebo control 4 mL; or active (73 mg or 146 mg) in a 1:1:4:4 ratio.

A randomization scheme will be used to program an Interactive Web-based Response System (IWRS). If considered eligible, subjects will be randomly assigned to a treatment group on Day 1 using this system. When a subject is randomized, the IWRS will assign a randomization number for each subject, corresponding to a specific treatment code. The randomization number will be automatically input into the eCRF by the system. The corresponding treatment code will only be accessible to the unblinded personnel.

For each drug distribution visit, the randomization number and the study treatment code assigned to a given subject, will then be used to select the appropriate bottle of blinded study drug for that subject for the corresponding visit.

The randomization scheme and corresponding treatment code will be kept strictly confidential, accessible only to authorized persons, until the time of unblinding. If a subject discontinues from the study, neither the subject identification number nor the allocated randomization code will be reused, and the subject will not be allowed to be re-randomized to the study.

8.2 Subject Informed Consent

Written consent will be obtained from the subject prior to any study-specific procedure or investigation.

Information about the study will be given to the subject both verbally and in writing in a language (eg, English) that is understood by the subject. The written subject information will explain the objectives of the study and its potential risks and benefits. The subject should have adequate time to read the information and to ask the Investigator any questions. The Investigator must be satisfied that the subject has understood the information provided before written consent is obtained. If there is any doubt as to whether the subject has understood the written and verbal information, the subject should not enter the study.

If a subject agrees to participate, then he/she will be asked to sign and date an ICF, the original of which will be kept by the Investigator. A copy of the signed ICF will be given to the subject. A record should also be made in the notes that the subject voluntarily agreed to participate in the study and the original, signed ICF must be made available to the study monitor for inspection.

8.3 Study Visits

8.3.1 Scheduled Visits

For each subject, a maximum of 10 scheduled visits will be performed. The procedures and assessments to be performed during each period/time point are indicated in the Schedule of Assessments. Further relevant details of the evaluations to be performed at each visit are described below.

Screening Period (Day -45 to Day -28)

- Written informed consent will be obtained.
- Subject eligibility will be determined based on IENFD score, medical history, physical examination, height and weight, review of entry criteria, 12-lead ECG, clinical laboratory tests (including serum pregnancy test for females), hepatitis/HIV screening, and drug/alcohol/urine screening.

Baseline Period (Predose Day 1/Baseline)

- On day 1, the subject will visit the clinical unit and will be evaluated to ensure that he/she meets entry criteria. The subject should arrive fasted (12 hours).
- On day 1, the subject will have the baseline assessments and safety laboratory evaluations performed as indicated in the Schedule of Assessments.
- Assessments for AEs will occur throughout the baseline period.

Treatment Period (Day 1 through Week 24)

- The subject will receive the investigational product and will be trained on how to apply the material on day 1.
- Blood (a single 2 mL sample) will be collected for PK trough levels, at weeks 2, 4, 8, 12, 16, 20, and 24..
- Pharmacodynamic and efficacy evaluations will be performed as indicated in the Schedule of Assessments.
- Blood for clinical laboratory tests will be drawn after an overnight fast (12 hours) at weeks 2, 4, 8, 12, 16, 20, and 24.
- Assessments for AEs and queries for concomitant medication usage will occur throughout the treatment period.

Post-treatment Follow-up Visit (Week 26)

- The subject will be assessed for AEs and queried for concomitant medication usage since previous visits.
- Symptom directed physical exam.

- Biopsy examination and suture removal, if necessary.

8.3.2 Unscheduled Visits

Should there be need for an unscheduled visit, (eg, to manage or follow up on unresolved AEs), the Investigator should use their judgment and perform an adequate evaluation of the subject. Vital signs, physical examination, and ECG findings should be performed as necessary and the data recorded in the Unscheduled Visit section of the eCRF.

8.4 General Restrictions

8.4.1 Fasting Treatment Periods

On all visit days during which clinical laboratory samples will be obtained, subjects will have fasted for approximately 12 hours (overnight) before sample collection.

8.4.2 Smoking

Smoking is not prohibited during the trial. However, a stable nicotine intake regimen is required 3 months prior to participation in the trial and throughout the duration.

8.4.3 Physical Exercise

No change from prestudy exercise routine is requested.

8.4.4 Contraceptive Measures

The subject or their heterosexual partner (if applicable) should be surgically sterile (vasectomy, hysterectomy, bilateral tubal ligation [both tubes tied], or bilateral oophorectomy [both ovaries removed]). If not, and the female subject or partner is of childbearing potential (ie, could become pregnant), the subject must agree to use highly effective contraception during heterosexual intercourse throughout the study period and for 1 month following the last dose of the study drug.

8.4.4.1 Acceptable Forms of Contraception

A highly effective method of birth control is defined as those that result in a low failure rate (ie, less than 1% per year) when used consistently and correctly **When used individually, hormonal, barrier, or IUD methods alone are not acceptable**.

Examples of acceptable forms of highly effective contraception include the following:

- Established use of oral, injected, or implanted hormonal methods of contraception in combination with a barrier method(e.g, combined oral contraceptives, patch, vaginal ring, injectables, and implants).
- Placement of an IUD or intrauterine system in combination with a barrier method.

- Sterilized male partner (with the appropriate post-vasectomy documentation of the absence of sperm in the ejaculate) in combination with a barrier method.
- True abstinence: When this is in line with your preferred and usual lifestyle.

Examples of nonacceptable methods of contraception include:

- Barrier method alone (male condom, female condom, contraceptive sponge, cervical cap, diaphragm, etc.)
- Periodic abstinence (eg, calendar, ovulation, symptothermal, post-ovulation).
- Withdrawal.
- Spermicide.

However, if a form of highly effective method of birth control is not achievable, then a combination of at least two of the barrier methods listed above may be used (e.g. male condom with diaphragm, male condom with cervical cap, etc).

8.5 Compliance with the Protocol

The Investigator will agree to implement the study protocol as written and adhere to the guidelines stated in the Investigator's statement, which will be signed by the Investigator prior to the start of the study. The study will be performed in accordance with the Declaration of Helsinki, International Council for Harmonisation (ICH) Good Clinical Practice (GCP) guidelines, and Health Canada regulations.

Any noncompliance to the protocol should be discussed with WinSanTor. All deviations from the regimen defined above should receive written approval from the study medical monitor, must be explained, and the reason entered on the CRF. WinSanTor retains the right to require the withdrawal of any subject who violates the protocol.

8.6 Termination of the Study

If, in the opinion of the Investigator, the clinical observations in the study suggest that it may be unwise to continue, the Investigator may terminate the study after consultation with WinSanTor. A written statement fully documenting the reasons for such a termination will be provided to the sponsor. In addition, the sponsor may terminate the study at any time.

Furthermore, if it becomes apparent that subject enrollment is unsatisfactory with respect to quality or quantity, or that data recording is inaccurate or incomplete on a chronic basis, the sponsor has the right to terminate the study and remove all study materials from the investigational site. A written statement will be provided to the Investigator, the IRB, and regulatory authorities, if required. In the event that any SAEs are reported as part of the reason for early termination of the study, all documentation relating to the event(s) reported to the Agency must be obtained and filed appropriately.

9 STUDY MEDICATION

9.1 Description of Study Medication

The study drug will be manufactured, tested and bulk packaged, under Good Manufacturing Practice (GMP) by Tergus Pharma, Durham NC, USA. The bulk packaged bottles will then be sent to Xerimis, Inc. Moorestown, NJ, USA to be blind labeled under GMP. Xerimis will then ship the material to a central depot in Canada (Ropack Pharma Solutions, Montreal, Canada) and from there the bottles will be directly shipped to the study sites.

The active pharmaceutical ingredient in WST-057 is pirenzepine free base monohydrate. The physical, chemical, and pharmaceutical properties of pirenzepine free base monohydrate are presented in Table 2.

**Table 2. Physical, Chemical, and Pharmaceutical Properties of Pirenzepine Free Base Monohydrate**	
**Name:**	Pirenzepine free base monohydrate
**Chemical classification:**	M_1 muscarinic receptor antagonist
**Chemical name:**	11-[2-(4-methylpiperazin-1-yl)acetyl]-5H-pyrido[2,3-b] [1,4]benzodiazepin-6-one monohydrate
**Chemical formula:**	$C_{19}H_{21}N_5O_2*H_2O$
**Structural formula:**	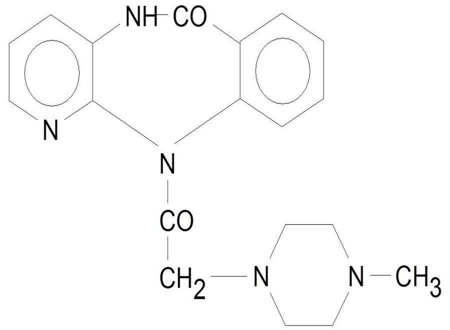 $*H_2O$
**Molecular weight:**	369.6 g/mol
**CAS Reference number:**	28797-61-7
**Appearance:**	White or yellowish, crystalline powder
**Solubility:**	Slightly soluble in water and methanol, very slightly soluble in ethanol<br>Practically insoluble in methylene chloride

The pirenzepine free base monohydrate is manufactured by Archimica S.p.A. (previously known as Euticals S.p.A) (Italy) in accordance with GMP. Pirenzepine free base is obtained through the condensation of N-methylpiperazine with 5,11 dihydro 11 chloroacetyl 6H pyrido [2,3 b][1,4]benzodiazepin 6 1 (DCPB). The pirenzepine base is then purified and salified with HCl in aqueous acetone to produce pirenzepine dihydrochloride monohydrate. The pirenzepine free base monohydrate is made by taking the GMP pirenzepine dihydrochloride and solubilizing the material in a sodium hydroxide solution, washing with pure water and then dried.

The drug product is a low-viscosity, clear colorless solution. Table 3 provides the composition of WST-057.

Table 3. Composition of Pirenzepine and Placebo Topical Solutions

Component and Quality Standard	Function	Strength/Label Claim (mg)			
		2 mL (73 mg active/day)	4 mL (146 mg active/day)	2 mL Placebo	4 mL Placebo
Pirenzepine free base anhydrous	Active	73.2	146.4	0.0	0.0
Dimethyl sulfoxide, USP	Penetration enhancer	292.6	585.2	292.6	585.2
Capric/caprylic triglyceride,NF, croda GTCC	Solubilizer and emollient	365.0	730.0	438.2	876.4
Laureth-4, (Ph Eur), brij L4	Emulsifier	55.0	110.0	55.0	110.0
Ethanol, USP	Solvent/ penetration enhancer/ preservative	740.8	1481.6	740.8	1481.6
Propylene glycol, USP	Solvent	274.3	548.6	274.4	548.8
Hydroxypropyl cellulose (HY119), NF	Polymer/ thickening agent	27.5	55.0	27.4	54.8
BHT, NF	Anti-oxidant	1.83	3.66	1.84	3.68
Total (mg)		1830.23	3660.46	1830.24	3660.48

BHT = butylated hydroxytuluene; NF = National Formulary; Ph Eur = European Pharmacopoeia; USP = United States Pharmacopoeia.

9.2 Drug Packaging

Subjects will be provided with a labeled, airless metered dosing pump (145 mL total volume), made of medical-grade polypropylene plastic, filled with either WST-057 solution or placebo solution on visit day 1 and at weeks 4, 8, 12, 16, and 20.

9.3 Drug Labeling

Each bottle of investigational product will be labeled with (at least) the following information:

- Study number
- Name and address of the sponsor and manufacturer

- Drug name (or placebo), dosage form, route of administration, strength, and quantity of drug per bottle
- Identifying code (eg, bottle number) for blinded studies
- Lot number
- Directions for use, including required volume to dispense for correct dosage
- The statement, "Caution—New Drug—Limited by Federal Law to Investigational Use"
- Storage conditions
- Expiry date

9.4 Drug Storage

All study medication will be stored up until 1 month prior to the expiry date in the refrigerator (2-8ºC) in a secured area where access is limited to the Investigator and his/her staff.

9.5 Dispensing and Administration of Treatment

The drug product and corresponding matched placebo will be shipped to the site. All material (labeled metered pumps) will be checked in by the unblinded pharmacist and stored under the appropriate conditions.

Subjects will be randomly allocated in a 1:1:4:4 ratio into placebo 2 mL, placebo 4 mLor one of 2 doses of WST-057 (2.0mL or 4.0 mL). A randomization scheme will be used to program an Interactive Web-Based Response System (IWRS). Eligible subjects will be randomly assigned to a treatment group on Day 1. The randomization number and the study treatment code assigned to a given subject by will be used to select the appropriate bottle of blinded study drug for that subject for the corresponding visit.

Subjects will be instructed on topical administration of 2 mL WST-057 (73 mg pirenzepine free base monohydrate), or 4 mL WST-057 (146 mg pirenzepine free base monohydrate), or 2 mL or 4 mL of placebo based on randomization. The metered pumps are set to deliver 1 mL per pump. Therefore, the investigational product should be administered by the subject by dispensing 2 or 4 pumps of material (placebo or active) into the palm of their hand and spreading evenly over the calves (mid-calf sock line), ankles, and tops and bottoms of feet. After application, subjects will be instructed to allow the material to dry, prior to covering the dosing area with socks or other soft dressing. Therefore, investigational material should be applied during a convenient time of the day, eg, administer drug product in the evening and allow it to dry prior to going to bed. Administration is to be done at approximately the same time every day. The subject will be instructed to store the study drug at refrigerated temperature. The study drug is not to be stored in direct sunlight or left in an automobile. However, the subject may store the IP near their bedside at room temperature in order to support and improve compliance.

9.6 Drug Accountability

The Investigator or their designee will sign a receipt for the study drugs on the day they are delivered to the study site. A copy of this receipt must be filed in the Investigator study file and the original returned as directed. A drug accountability record should be maintained by the unblinded pharmacist responsible for dispensing the study drug to the subject. This should record what supplies are issued to which subjects. WinSantor or its designee should be notified immediately of details of any supplies that are inadvertently damaged. Details of any supplies that are inadvertently damaged or unaccountable for any reason should be noted on this drug accountability record, which will be collected by WinSanTor or designee at the end of the study. The contracted research clinic will be responsible for drug accountability at their research clinic.

All drugs will be inventoried by WinSanTor or designee during and at the conclusion of the study. Secure disposal or return of unused supplies to the sponsor at the end of the study will be arranged.

9.7 Procedure for Unblinding

The randomization code may be broken when this knowledge will affect the management of an urgent medical event. If possible, before the unblinding of a given subject during the treatment period, the medical monitor should be contacted to discuss the case before the code is broken.

An emergency unblinding feature will be provided within the IWRS. Each site will designate a person who can conduct the emergency unblinding feature within the system. The person delegated will be given special permissions and a unique password that will allow them to unblind subjects. Outside of this delegate, the randomization schedule will not be revealed to study subjects, investigators, clinical staff, or site managers until the database is locked.

If it becomes necessary to unblind treatment information during the study, the reason for unblinding is to be documented in the eCRF. The Investigator is to contact the sponsor promptly and explain the reason for any premature unblinding (eg, unblinding due to an SAE) and document this reason appropriately.

10 CONCOMITANT MEDICATION

10.1 General Considerations

The Investigator is to record the use of all concomitant medications, both prescribed and over-the-counter, in the eCRF. Any medication must have been stopped as required in the inclusion and exclusion criteria prior to the first dose of study drug. Subjects should be advised against taking any new medication, both prescribed and over-the-counter, without consulting the Investigator, unless the new medication is required for emergency use. On day 1, the study site personnel should ensure that subjects continue to meet the inclusion criteria and none of the exclusion criteria (including no prohibited medications).

10.2 Prohibited Medication

The use of any medications other than study drug or other stable, permitted medications is prohibited during the trial, except as necessary to treat AEs under the direction of the Investigator.

Episodic or periodic treatments for pain, such as monthly injections, are not permitted.

10.3 Permitted Medication

Patients may be on a stable (ie, > 4 weeks at the same dosage) antidiabetic treatment (eg, insulin, oral agents, and/or lifestyle) and/or analgesic treatment and/or nonpharmacologic pain treatment. All stable regimens must have been in place for a minimum of 4 weeks and be expected to remain the same for the duration of the study, unless a medial reason requires modification. Nonpharmacologic pain treatments include the following: relaxation/hypnosis, physical or occupational therapy, and counseling.

11 ADVERSE EVENTS

11.1 Adverse Event Definition

An AE is any untoward medical occurrence in a subject administered a pharmaceutical product, which does not necessarily have to have a causal relationship with the pharmaceutical product treatment. An AE can, therefore, be any unfavorable and unintended sign, symptom, or disease temporally associated with the use of a medicinal product, whether or not related to the medicinal product. Any worsening of the subject's disease under study or other medical conditions will also be considered to be an AE, unless it is within the normal range of disease fluctuation for that subject.

Clinically meaningful (for a given subject) changes in physical examination findings and abnormal objective test findings (eg, laboratory, X-ray, or ECG) should also be recorded as AEs. The criteria for determining whether an abnormal objective test finding should be reported as an AE are as follows:

1. Test result is associated with accompanying symptoms or is of clinical concern.
2. Test result requires additional diagnostic testing or medical/surgical intervention.
3. Test result leads to a change in study dosing or discontinuation from the study, significant additional concomitant drug treatment, or other therapy.
4. Test result leads to any of the outcomes included in the definition of an SAE.

Note that merely repeating an abnormal test, in the absence of any of the above conditions, does not meet condition 2 above for reporting as an AE.

Any abnormal test result that is determined to be an error does not require reporting as an AE.

11.2 Reporting of Adverse Events

At each evaluation, the Investigator will determine whether any AEs have occurred. The subject will be questioned in a general manner and no specific symptoms will be suggested. If any AEs have occurred, they will be recorded in the subject's source documents and on the appropriate pages of the eCRF. If known, the diagnosis should be recorded, in preference to the listing of individual signs and symptoms.

AE reporting begins when the subject signs the ICF and ends at the conclusion of the post-treatment follow-up period unless an unresolved AE is still being followed (addressed in further detail in section 11.3).

The intensity of the AE will be graded as follows:

Mild:	The AE is easily tolerated and does not interfere with normal daily activities.
Moderate:	The AE causes some interference with daily activities.
Severe:	The AE causes all normal daily activities to be completely halted.

The Investigator will make a judgment regarding whether or not the AE was related to study drug, as outlined below. The Investigator will evaluate any changes in laboratory values, make a determination as to whether or not the change is clinically important, and whether or not the changes were related to study drug. However, even if the Investigator believes there is no relationship to the study drug, the AE or laboratory abnormality **MUST** be recorded in the eCRF.

The following is how the relationship of the AE to study treatment will be addressed:

None:	The AE must definitely be caused by the subject's clinical state or the study procedure/conditions (ie, it has no association with the study drug).
Improbable:	The temporal association between the AE and the study drug is such that the study drug is not likely to have any reasonable association with the AE.
Possible:	The AE follows a reasonable temporal sequence from the time of drug administration, but could have been produced by the subject's clinical state or the study procedures/conditions.
Probable:	The AE follows a reasonable temporal sequence from the time of study drug administration, abates upon discontinuation of the study drug, and cannot be reasonably explained by the known characteristics of the subject's clinical state.
Definite:	The AE follows a reasonable temporal sequence from the time of study drug administration, abates upon discontinuation of the study drug, and reappears when the study drug is introduced.

The Investigator will record the action taken and outcome for each AE according to the following:

Action Taken

- None
- Treatment required
- Hospitalization
- Subject withdrawn
- Administration of study drug temporarily interrupted
- Administration of study drug permanently discontinued
- Other (specify)

Outcome

- Recovered
- Improved
- Ongoing
- Death
- Lost to follow-up

11.3 Follow-Up of Adverse Events

If any AEs are present when a subject completes the study or if a subject withdraws or is withdrawn from the study, the subject will be re-evaluated within 2 weeks, when possible. At the Investigator's discretion, minor AEs can be re-evaluated via telephone and documented. If the AE has still not resolved, additional follow-up will be performed as appropriate. Every effort is to be made by the Investigator or delegate to contact the subject until the AE has resolved or stabilized or the medical monitor and Investigator agree that further follow-up is not necessary. This should be documented in the subject's source documents, eCRF, and as necessary, for AE outcome and resolution.

12 SERIOUS ADVERSE EVENTS

An SAE is any untoward medical occurrence that, at any dose:

- Results in death
- Is life-threatening
- Requires subject hospitalization or prolongation of existing hospitalization
- Results in persistent or significant disability/incapacity
- Is a congenital anomaly/birth defect

Medical and scientific judgment should be exercised in deciding whether expedited reporting is appropriate in other situations, such as important medical events that may not be immediately life-threatening or result in death or hospitalization, but may jeopardize the subject or may require intervention to prevent one of the other outcomes listed in the definition above. These should also be considered to be SAEs. The site will report SAEs to the sponsor and pharmacovigilance within 24 hours of notice in accordance with the safety plan.

12.1 Life-threatening Adverse Event

The term "life-threatening" in the definition of "serious" refers to an event in which the subject was at risk of death at the time of the event; but it does not refer to an event which, hypothetically, might have caused death if it were more severe.

12.2 Hospitalization

This is defined as the subject being hospitalized overnight, or the subject's hospital stay being prolonged for at least an additional overnight stay. Preplanned hospital stays or hospital stays for nonmedical social reasons will not be considered as hospitalization. "Twenty-three hour hospitalizations" for observation should be discussed with the medical monitor to determine whether they are appropriate for SAE reporting.

12.3 Pregnancy

Female subjects must be surgically sterile (hysterectomy, bilateral oophorectomy, or bilateral tubal ligation) **or** if a female subject is of childbearing potential, she must agree to use a medically acceptable, highly effective method of contraception during the study and for 1 month after the last dose of study drug to be eligible for participation in the study (see Inclusion Criteria, section 6.2; and Contraceptive Measures). All female subjects must have a negative serum pregnancy test at screening and a negative urine pregnancy test at screening day and day 1 to be eligible for the study. A urine pregnancy test will also be performed at the end of the study at the post-treatment follow-up visit.

12.3.1 Pregnancy Follow-up

Female subjects should be instructed to notify the Investigator if pregnancy is discovered either during or within 4 weeks of completing the last dose. The pregnancy event will be reported in the same reporting time frame as an SAE (section 12.5) on a specific Pregnancy Report Form, and the status of mother and child will be reported to the sponsor as soon as possible after delivery.

Should a female become pregnant or suspect she is pregnant while participating in this study, or should a male subject's partner become pregnant or suspect she is pregnant while the male is participating in this study, the treating Investigator should be informed immediately. Female subjects will be withdrawn from the study, and all pregnancies will be reported along the same timelines as an SAE.

If a female subject or the female partner of a male subject who has received pirenzepine becomes pregnant, the pregnancy will be recorded. The sponsor and the sponsor's pharmacovigilance group will be informed. The subject will be asked to provide information. The pregnancy will be followed to term and any premature terminations or obstetric complications reported on the outcome of the pregnancy, including premature termination, should the case arise.

Spontaneous miscarriage and congenital abnormalities will be reported as an SAE. All pregnancies involving potential exposure to pirenzepine will be followed to term, and the health status of mother and child will be reported following delivery.

12.4 Subject Withdrawal

If the subject discontinues study treatment due to an AE and withdraws from the study, the reason for the subject withdrawal is to be recorded as due to a specific AE.

12.5 Reporting Serious Adverse Events

ANY SAE, WHETHER OR NOT RELATED TO THE STUDY TREATMENT, MUST BE REPORTED IMMEDIATELY TO THE SPONSOR'S MEDICAL MONITOR AT THE FOLLOWING NUMBERS:

PRA Drug Safety Centre
Fax: +44 1792 525 720
E-mail: MHGSafety@prahs.com

WinSanTor or designee will provide details to the Investigator of all serious, unexpected, and related AEs (or other events depending on the specific requirements) from any other ongoing clinical trials that should be reported to IRBs, as appropriate. Confirmation that these serious, unexpected, and related AEs have been submitted to the IRB must be forwarded to WinSanTor or designee within 24 hours of knowledge and also be kept in the Investigator files. The site will report SAEs and AEs to their local IRB in accordance with the reporting guidelines of the IRB/EC.

Fatal or life-threatening, unexpected ADRs occurring in clinical investigations qualify for very rapid reporting. Regulatory agencies should be notified (e.g., by telephone, facsimile transmission, or in writing) as soon as possible but no later than 7 calendar days after first knowledge by the sponsor that a case qualifies, followed by as complete a report as possible within 8 additional calendar days. This report must include an assessment of the importance and implication of the findings, including relevant previous experience with the same or similar medicinal products.

Serious, unexpected reactions (ADRs) that are not fatal or life-threatening must be filed as soon as possible but no later than 15 calendar days after first knowledge by the sponsor that the case meets the minimum criteria for expedited reporting.

13 STATISTICS

No formal power analysis was conducted. However, the population size and duration of the study was based on a similar study designed to observe a positive change in the Norfolk-QOL-DN measurement in 60 subjects with T2DM after 6 months treatment with topiramate and ruboxistaurin (Boyd et al, 2011).

13.1 Methods and Data Analysis

13.1.1 Sample Size Justification

No formal power calculations were performed to estimate appropriate group sizes since this is the first clinical evaluation of WST-057. Cohort size was selected to achieve an appropriate balance between unnecessarily exposing a large number of subjects to a novel therapeutic regimen, having sufficient subjects enrolled to interpret pharmacodynamic data, and making a reasonable assessment of the dose-related safety of WST-057.

13.1.2 Study Populations

Study populations will consist of the safety population, the intent-to-treat (ITT) population, and the per-protocol (PP) population. The safety population will consist of all subjects receiving at least 1 dose of study drug, and will reflect actual treatment received. The ITT population will include all subjects randomized to receive study drug according to randomized treatment who have at least 1 post-baseline measurement. The PP population will include subjects who have at least 80% drug compliance and complete all study visits.

13.1.3 Demographic/Baseline Information

Demographic/baseline information will be formally compared between treatment groups and will be summarized within each cohort by treatment group using descriptive statistics. For continuous variables, mean, standard deviation, median, minimum, and maximum will be presented. For categorical variables, N and % will be presented.

13.1.4 Analysis of Safety Data

The safety end point data will be summarized for the safety population.

The AEs will be coded using the latest version of Medical Dictionary for Regulatory Activities (MedDRA). The incidence of AEs will be summarized by body system and preferred term. A general summary of the AEs and SAEs will be summarized by overall number of AEs, and their intensity and relationship to study drug, per treatment group. The number of AEs leading to withdrawal or SAEs leading to death will also be summarized. The World Health Organization Drug Dictionary Enhanced (WHO-DDE) will be used to classify concomitant mediation by therapeutic class and drug name.

The safety laboratory data will be summarized by visit and by treatment group, along with the change from baseline. The values that are below the lower limit or above the upper limit of the reference range will be flagged. Those values or changes in values, which are identified as being clinically significant, will be flagged and summarized separately (with a supporting subject level listing).

The vital signs will also be summarized by visit and by treatment group, along with the change from baseline.

The ECG data will be summarized by standard parameter by time point/visit and by treatment group, along with the change from baseline.

Comparisons will be made between subjects receiving placebo and WST-057 at each dose level. Comparisons will be made among the dose levels, where appropriate. Nonparametric or parametric statistical methods will be used to make treatment group comparisons, where appropriate.

13.1.5 Interim Analysis

No formal interim analysis is planned for this study. Given the nature of the study design, the safety results will be evaluated as indicated elsewhere in this protocol by appropriately qualified individuals.

13.1.6 Pharmacokinetic Analysis

Trough blood samples will be collected and analyzed for WST-057 using a validated LC/MS/MS method. Full details of the methodology will be presented in the bioanalytical report, which will be appended to the clinical study report. The data obtained from these analyses will be used to calculate C_{min}, λ_z, $t_{1/2}$, CL/F, V_z/F, and renal clearance (CL_r). Further analyses details will be provided in the statistical analysis plan for this study.

13.1.7 Pharmacodynamic Analysis

The changes in IENFD between the baseline and terminal samples for each subject will be calculated and within-group change will be tested using a 2-tailed paired t test, with any result

with a $P < .05$ being considered significant. Analysis of changes from baseline to end of study results for the Norfolk-QOL-DN (and subset score analysis), Toronto Clinical Neuropathy Score, modified Toronto Clinical Neuropathy Score, quantitative thermal threshold, and quantitative vibrations perception will be outlined in the statistical analysis plan.

14 STUDY MANAGEMENT AND DATA COLLECTION

14.1 Ethical Conduct of the Trial

This study will be conducted according to the protocol; 21 CFR parts 50, 54, 56, and 312; the Declaration of Helsinki; and ICH GCP. The Investigator will conduct the trial according to applicable local laws and federal regulatory requirements.

14.2 Institutional Review Board

IRBs must be constituted according to the applicable state and federal requirements (including ICH GCP) of each participating location.

It is the responsibility of the clinical site to submit the protocol, IB, subject ICF, subject recruitment materials (if applicable), and other documentation (as required by the IRB) to its IRB for review and approval. A copy of the written approval must be provided to WinSanTor or designee. The documentation should clearly mention the approval/favorable opinion of the protocol, the subject ICF, and subject recruitment materials (if applicable), including respective version dates. The IRB written approval and a list of the voting members, their titles or occupations, and their institutional affiliations (or the name of the IRB chairperson and Federal Wide Assurance number) must be obtained from the IRB and provided to WinSanTor or designee prior to the release of clinical study supplies to the investigational site and commencement of the study. If any member of the IRB has direct participation in this trial, written notification regarding his or her abstinence from voting must also be obtained.

The clinical site must adhere to all requirements stipulated by its IRB. This includes notification to the IRB regarding: protocol amendments, updates to the subject ICF, recruitment materials intended for viewing by subjects, investigational new drug safety reports, serious and unexpected AEs, reports and updates regarding the ongoing review of the trial at intervals specified by the IRB, and submission of final study reports and summaries to the IRB.

14.3 Subject Informed Consent

Prior to any study-specific procedures being performed, subjects and persons conducting the consent discussion will be required to sign and date the IRB-approved ICF, and each subject will be given a copy. In addition, this information should be recorded in the subject's medical record (source document).

The written consent document will embody the elements of informed consent as described in the Declaration of Helsinki, 21 CFR part 50.25, ICH GCP, and in accordance with any local regulations. The Investigator is responsible for the preparation, content, and IRB approval of the informed consent. The ICF must be approved by the clinical site's IRB and be acceptable to WinSanTor or designee.

The ICF must be written in a language fully comprehensible to the prospective subject. The Investigator or designee shall give the subject adequate opportunity to read the ICF before it is signed and dated. Information will be given in both oral and written form, whenever possible,

and in the manner deemed appropriate by the IRB. Subjects must be given ample opportunity to inquire about details of the study.

14.4 Amendments to the Protocol

An amendment must be agreed to in writing by WinSanTor or designee and submitted to the Agency as an IND amendment. It must also be submitted to and approved by the IRB for the clinical site before the amendment can be implemented. Written approval of a protocol amendment is not required prior to implementation of changes to the protocol that eliminate immediate hazard to the subject; however, approval must be obtained as soon as possible thereafter. Any agreed amendments must also be signed by the Investigator.

14.5 Study Initiation

The Investigator must not enroll any subjects prior to the completion of a formal initiation meeting conducted by a representative of WinSanTor or designee. This meeting will include an inventory of study supplies and a detailed review of the study protocol and associated CRFs. The Investigator will not be supplied with study drug until all of the required regulatory documentation has been provided to WinSanTor or designee.

14.6 Study Monitoring

It is the responsibility of the Investigator to ensure that the study is conducted in accordance with the protocol, ICH GCP, CFR, and other applicable regulatory requirements, and the current Declaration of Helsinki, and that valid data are entered into the CRFs.

To achieve this objective, the study monitor's duties are to aid the Investigator and, at the same time, the sponsor, in the maintenance of complete, legible, well-organized, and easily retrievable data. The study monitor will review the protocol with the Investigator. In addition, the study monitor will explain the Investigator's reporting responsibilities and all applicable regulations concerning the clinical evaluation of the study drug.

The Investigator will permit the representatives of WinSanTor or designee to monitor the study as frequently as WinSanTor (or designee) deems necessary to determine that data recording and protocol adherence are satisfactory. It is estimated that for this study, a site monitoring visit will be conducted for study initiation, after each cohort is completed, and at study termination. The CRFs and related source documents will be reviewed in detail by the study monitor at each visit, in accordance with relevant SOPs and ICH GCP regulations. This includes results of tests performed as a requirement for participation in this study and any other medical records required to confirm information contained in the CRFs, such as past medical history and secondary diagnoses. The Investigator and his/her staff will be expected to cooperate with the study monitor and provide any missing or incomplete information whenever possible.

All monitoring activities will be reported and archived. In addition, monitoring visits will be documented at the investigational site by signature and date on the study-specific monitoring log.

14.7 Case Report Form

The eCRFs will be provided by validated electronic data capture screens. All subject data generated during the study will be recorded on the source documents and transcribed to the electronic data capture screens through a Web browser for entry into a validated electronic database by way of a secure internet connection.

The Investigator and clinical research associate (CRA) will ensure that all data are entered promptly, completely, and accurately and conform to source documents, in accordance with specific instructions accompanying the eCRFs. Any corrections will be recorded by an audit trail. The audit trail will include the user ID of the person making the change and the date the change was made and an explanation why the change was made.

The study monitor will verify the entered data against the source document and issue manual queries. The query text will be manually entered by the monitor. These queries will then be available for the site entry personnel to respond.

WinSanTor will only consider eCRFs to be complete when each eCRF has been reviewed and approved by the Investigator, indicating their assurance of the accuracy of all recorded data. It is expected that the Investigator and his/her staff will cooperate with the monitoring team and provide any missing data in a timely manner.

Laboratory data will be collected electronically and transferred to the CRO handling the eCRF data for integration with the eCRF data.

The 12-lead ECGs will be digitally recorded after a 10-minute rest in the supine position. All ECGs will be over-read by an appropriately qualified physician and the data entered in the subject’s eCRF or via validated direct electronic transfer for integration with the eCRF data.

For both of these datasets, a data format will be agreed upon between the CRO handling the clinical study data and the laboratory and ECG vendors. The data management programming group at the CRO will reconcile the external data with eCRF data. Discrepancies will be first addressed with the lab or ECG vendors. If data changes are required to the database, then queries will be issued. If external data need to be modified, the laboratory or ECG vendor will provide updated data.

14.8 Verification Procedures

In fulfillment of their obligations to WinSanTor and to verify compliance with this protocol, ICH GCP, and the CFR, the Investigator is required to authorize direct access to the subject’s medical records by WinSanTor or designee, IRBs, and regulatory authorities.

It is the Investigator’s obligation to ensure documentation of all relevant data in the subject’s source document record. The subject’s original data may not be recorded directly into the eCRF, but documented in source: medical history/concomitant disease, subject ID, confirmation of informed consent and the date of study enrollment, visit dates, administration of study medications, AEs (start and stop dates), and all concomitant medications (start and stop dates).

The Investigator will maintain a subject identification code list to enable unambiguous identification of each subject's name and corresponding subject number. The subject identification code list is an essential document and will be maintained according to ICH GCP guidelines.

14.9 Retention of Records

All documentation pertaining to the study will be kept by WinSanTor or designee in accordance with Agency regulations.

The study site will maintain a study file, which should contain the IB; protocol; drug accountability records; study correspondence; and other study-related documents to establish that the clinical trial is conducted in accordance with GCPs and the Regulations.

The sponsor shall maintain complete and accurate records in respect of the use of a drug in a clinical trial, including: a copy of all versions of the IB for the drug; records respecting each change made to the IB, including the rationale for each change and documentation that supports each change; records respecting all adverse events in respect of the drug that have occurred inside or outside Canada, including information that specifies the indication for use and the dosage form of the drug at the time of the adverse event; records respecting the enrolment of clinical trial subjects, including information sufficient to enable all clinical trial subjects to be identified and contacted in the event that the sale of the drug may endanger the health of the clinical trial subjects or other persons; records respecting the shipment, receipt, disposition, return and destruction of the drug; for each clinical trial site, an undertaking from the qualified investigator that is signed and dated by the qualified investigator prior to the commencement of his or her responsibilities in respect of the clinical trial, that states that: the qualified investigator will conduct the clinical trial in accordance with GCPs, and the qualified investigator will immediately, on discontinuance of the clinical trial by the sponsor, in its entirety or at a clinical trial site, inform both the clinical trial subjects and the REB of the discontinuance, provide them with the reasons for the discontinuance and advise them in writing of any potential risks to the health of clinical trial subjects or other persons; for each clinical trial site, a copy of the protocol, ICF and any amendment to the protocol or ICF that have been approved by the REB for that clinical trial site; and for each clinical trial site, an attestation, signed and dated by the REB for that clinical trial site, stating that it has reviewed and approved the protocol and ICF and that the board carries out its functions in a manner consistent with GCPs. The sponsor shall maintain all records referred to in the applicable Regulations for a period of 25 years.

The Investigator agrees to keep records and those documents that include (but are not limited to) the identification of all participating subjects, medical records, study-specific source documents, source worksheets, all original signed and dated ICFs, copies of all CRFs, query responses, and detailed records of drug disposition, to enable evaluations or audits from regulatory authorities and WinSanTor or its designee(s).

The Investigator shall retain records required to be maintained under this part for a period of 25years. However, these documents should be retained for a longer period if required by the

applicable regulatory requirement(s) or if needed by the sponsor. In addition, the Investigator must make provision for the subject's medical records to be kept for the same period of time.

No data should be destroyed without the agreement of the sponsor. WinSanTor will inform the Investigator in writing when the trial-related records are no longer needed.

Subjects' original data will be archived in accordance with the archiving regulations or facilities of the investigational site.

14.10 Insurance and Indemnity

In the event that a subject suffers injury or death directly attributable to treatment with the investigational drug and/or as a result of any action taken under this protocol, appropriate treatment and/or compensation will be provided by and/or paid to the subject by WinSanTor in accordance with applicable federal and international laws and/or guidelines.

14.11 Audit

It is the responsibility of WinSanTor or designee to perform audits (if applicable) as part of implementing quality assurance. The purpose of an audit, which is independent of and separate from routine monitoring or quality control functions, is to evaluate trial conduct and compliance with the protocol, SOPs, ICH GCP, and the applicable regulatory requirements. The auditor and regulatory authorities will require authority from the Investigator to have direct access to the subject's medical records.

15 USE OF INFORMATION

15.1 General Aspects

All information concerning WinSanTor or designee, such as patent applications, formula, manufacturing processes, basic scientific data, or formulation information supplied by WinSanTor or designee and not previously published, is considered confidential and shall remain the sole property of WinSanTor. The Investigator agrees to use this information only in accomplishing this study and will not use it for other purposes without the written consent of WinSanTor, except for official representatives, such as regulatory authorities.

It is understood by the Investigator that the information derived from this clinical study, in connection with the development of WST-057, will be used by WinSanTor and, therefore, may be disclosed by WinSanTor as required to other clinical Investigators, other pharmaceutical companies, and other government agencies. In order to allow for the use of the information derived from clinical studies, it is understood that there is an obligation to provide to WinSanTor complete test results and all data compiled in this study.

15.2 Subject Confidentiality and Data Protection

WinSanTor and its designees affirm and uphold the principle of the subject's right to protection against invasion of privacy. Throughout this study, all data will be linked to the CRF via a unique identification number and the subject's initials. The data will be blinded correspondingly in all data analyses.

However, in compliance with Health Canada guidance and the ICH Guidelines, and in fulfillment of its obligations to WinSanTor to verify compliance with this protocol, WinSanTor and/or designee requires that the Investigator permit its study monitor, WinSanTor-designated auditors, IRBs, and other governmental regulatory authorities to review the subject's primary medical records (source data or documents), including but not limited to laboratory test result reports, ECG reports, admission and discharge summaries for hospital admissions occurring during a subject's study participation, and autopsy reports of deaths occurring during the study.

Should access to such medical records require a waiver or authorization separate from the statement of informed consent, the Investigator will obtain such permission in writing from the subject before the subject is entered into the study.

15.3 Final Report and Publication Policy

All information regarding this study shall be kept strictly confidential. All data derived from the study shall be the property of WinSanTor. The Investigator must not undertake submitting any part of the data from this study for publication without prior consent of WinSanTor. WinSanTor may disclose data derived from the study to other Investigators and drug regulatory authorities.

After completion of the study, the Investigator (upon agreement with WinSanTor) may prepare a publication and shall send a draft manuscript of the publication to WinSanTor to reach agreement between the Investigator and WinSanTor on the contents of the publication. The

Investigator shall receive written approval from WinSanTor prior to submission of the final version for publication.

At the conclusion of the study, after the data are analyzed, WinSanTor or their designee will prepare a final clinical study report.

16 REFERENCES

Beiswenger KK, Calcutt NA, Mizisin AP. Epidermal nerve fiber quantification in the assessment of diabetic neuropathy. *Acta Histochem.* 2008;110(5):351-362.

Boyd A, Casselini C, Vinik E, Vinick A. Quality of life and objective measures of diabetic neuropathy in a prospective placebo-controlled trial of ruboxistaurin and topiramate. *J Diabetes Sci Technol.* 2011;5(3):714-722.

Calcutt NA, Smith DR, Frizzi K, et al. Selective antagonism of muscarinic receptors is neuroprotective in peripheral neuropathy. *J Clin Invest*. 2017;127(2):608-622.

CDC. National Diabetes Fact Sheet. 2007. Atlanta, GA.

Dworkin RH, Turk DC, Wyrwich KW, et al. Interpreting the Clinical Importance of Treatment Outcomes in Chronic Pain Clinical Trials: IMMPACT Recommendations. *The Journal of Pain*. 2008; 9(2): 105-121.

Edwards JL, Vincent AM, Cheng HT, Feldman EL. Diabetic neuropathy: mechanisms to management. *Pharmacol Ther*. 2008;120(1):1-34.

Gastrozepin 50 mg Tablet Information for the Health Expert. Boehringer Ingelheim GmBH. 2013.

Hammer R, Koss FW. The pharmacokinetic profile of pirenzepine. *Scand J Gastroenterol Suppl*. 1979;57:1-6.

Lauria G, Hsieh ST, Johansson O, et al. European Federation of Neurological Societies/Peripheral Nerve Society Guideline on the use of skin biopsy in the diagnosis of small fiber neuropathy. Report of a joint task force of the European Federation of Neurological Societies and the Peripheral Nerve Society. *Eur J Neurol.* 2010;17(7):903-912.

Lauria G, Holland N, Hauer P, Cornblath DR, Griffin JW, McArthur JC. Epidermal innervation: changes with aging, topographical location, and in sensory neuropathy. *J Neurol Sci.* 1999;164(2):172-178.

Perrot S, Lanteri-Minet M. Patients' Global Impression of Change in the management of peripheral neuropathic pain: Clinical relevance and correlations in daily practice. *European Journal of Pain.* 2019;23:1117-1128.

Poulose S, Cheriyan E, Poulose A, Cheriyan R, Vadakkanezath B, Zeimer P. Usefulness of the NC-stat DPNCheck nerve conduction test in a community pharmacy as an educational tool for patients with diabetes. *Can Pharm J (Ott)*. 2015;148(1):17-20.

Tanswell P, Kasper W, Zahn G. Automated monoclonal radioimmunoassays for pirenzepine, a selective muscarinic receptor antagonist, in plasma and urine. *J Immunol Methods.* 1984;93(2):247-258.

Tesfaye S, Boulton AJ, Dyck PJ, et al. Diabetic neuropathies: update on definitions, diagnostic criteria, estimation of severity, and treatments. *Diabetes Care*. 2010;33(10):2285-2293.

APPENDICES

APPENDIX A: Draize Dermal Scale

Grade	Dryness	Erythema Scale	Pruritus	Burning/Stinging	Edema
0.0	No dryness	No erythema	No pruritus	No burning/stinging	No edema
0.5	Perceptible dryness, fine white lines				
1.0	Fine dry lines, white powdery look and/or some uplifting flakes on less than 30% of the test site	Very slight erythema (barely perceptible)	Occasional, slight itching (barely perceptible)	Slight warm sensation (barely perceptible)	Very slight edema (barely perceptible)
1.5	More uniform flaking, covering 30%-50% of the test site				
2.0	Uniform, marked flaking covering more than 50% of the test site area and/or isolated scaling	Well-defined erythema	Intermittent itching	Warm, sensitive skin; not really bothersome	Slight edema (edges of area well-defined by definite puffiness)
2.5	Slight to moderate scaling				
3.0	Moderate to severe scaling with some uplifting of the scales	Moderate to severe erythema	Constant itching/scratching which is not disturbing sleep	Definite hot/stinging sensation that is somewhat bothersome	Moderate edema (raised approximately 1 mm)
3.5	Severe scaling and/or slight fissuring				
4.0	Severe scaling and severe fissuring	Severe erythema (beet redness) to slight eschar formation (injuries in depth)	Bothersome itching/scratching which is disturbing sleep	Hot stinging sensation that causes definite discomfort	Severe edema (raised more than 1 mm and extending beyond the area of exposure)
