# Supplementary material for: Topical application of the antimuscarinic pirenzepine increased lower limb nerve fibre density in a phase 2a study in type 2 patients with diabetes with peripheral neuropathy
Source: eBioMedicine. 2025 Dec 5;123:106055. doi: 10.1016/j.ebiom.2025.106055 (PMC12721300; doi:10.1016/j.ebiom.2025.106055)
Supplement: Supplementary Material 2 [file mmc3.docx]

**Inclusion Criteria**

Subjects must meet all of the following inclusion criteria to be eligible to participate in the study:

1. Diagnosis of T2DM (as defined by the 2013 Diabetes Canada guidelines).

2. Male and female patients in the age range of 18 to 75 years (inclusive).

3. Presence of definite diabetic neuropathy (as defined by the Toronto Consensus Guidelines) of at least 12 months duration in the lower extremities.

4. Provide written informed consent prior to entering the study or undergoing any study procedures.

5. Females should be either not of childbearing potential as a result of surgery or menopause (1 year after onset), or of childbearing potential and practicing a medically acceptable method of contraception (eg, abstinence, a barrier method plus spermicide, or intrauterine device [IUD]) for at least 1 month before the screening visit and for 1 month after the end of the study. Eligible female subjects must also have a negative serum beta-human chorionic gonadotropin (ß-hCG) at the screening visit. Those females using hormonal contraceptives must also use an additional approved method of contraception (eg, a barrier method plus spermicide or IUD) starting with the baseline phase and continuing throughout study treatment and for 1 additional menstrual cycle following the end of treatment.

6. Males must use an acceptable form of contraception.

7. Sural nerve response must be present and at least 1 µV in amplitude.

8. Patients must have a screening IENF density range of no less than 1 IENF/mm and no more than 10 IENF/mm.

9. Participating subjects must be reliable, willing, and able to cooperate with all study procedures, including the following: • Return for study visits on the required dates • Be physically able to inspect calves, tops of ankles, and soles of feet for wounds, infections, or other anomalies, and be able to self-administer the investigational drug to calves and feet. • Be able to accurately and reliably report symptoms (including treatment-emergent signs and symptoms). • Take study drug as required by protocol.

10. Be on stable antidiabetic treatment (insulin, oral agents, or lifestyle) that is not anticipated to change during the course of the study, except if medically required.

11. Be on stable analgesic treatment (same medication and dose) or stable nonpharmacological pain treatment for at least 4 weeks prior to screening and remain on this stable treatment throughout the study (unless otherwise directed by a physician). Nonpharmacologic pain treatment includes the following: relaxation/hypnosis, physical or occupational therapy, counseling, etc. Episodic or periodic treatments, such as monthly injections for treatment of pain (eg, local anesthetics), will not be permitted.

12. General health status must be acceptable for participation in this 24-week clinical study, with no hospitalizations for medical conditions within 12 weeks before and during screening per judgment of the Investigator. Any question regarding eligibility will be addressed with the medical monitor.

13. Fluency (oral and written) in the language in which the standardized tests will be administered.

**Exclusion Criteria**

Subjects who meet any of the following exclusion criteria will be excluded from participating in the study:

1. Lower leg IENFD at screening of < 1 or >10 IENF/mm.

2. Sural nerve amplitude of < 1 µV at the ankle.

3. Proliferative retinopathy or maculopathy requiring acute treatment.

4. Requiring dialysis.

5. Impaired liver function, defined as aspartate aminotransferase (AST) or alanine aminotransferase (ALT) ≥ 3 times the upper limit of normal.

6. Presence of clinically significant peripheral or autonomic neuropathy that is clearly of nondiabetic origin.

7. Uncontrolled treated/untreated hypertension (systolic blood pressure [BP] ≥ 180 or diastolic BP ≥ 100 at screening).

8. Amputations of lower extremities or presence of foot ulcers.

9. Clinically significant active macrovascular disease, including myocardial infarction or cerebrovascular event within the past 12 months.

10. Uncontrolled or untreated hypothyroidism.

11. Active infection (eg, HIV, hepatitis), or a history of severe infection during the 30 days prior to screening.

12. Evidence of severely immunocompromised status.

13. Major surgical procedure during the 90 days prior to screening

14. Diagnosis and/or treatment of malignancy (except for basal cell or squamous cell skin cancer, in-situ carcinoma of the cervix, or in-situ prostate cancer) within the past 5 years.

15. Clinically significant gastric emptying abnormality (eg, severe gastroparesis).

16. Urinary retention or an enlarged prostate.

17. Uncontrolled glaucoma.

18. Other clinically significant, active (over the past 12 months) disease of the gastrointestinal, pulmonary, neurological, genitourinary, or hematological system that, in the opinion of the Investigator, would compromise the subject’s participation in the study, might confound the results of the study, or pose additional risk in administering the study drug.

19. New treatment with (< 3 months) antioxidant supplements or drugs known to affect oxidative stress and peripheral diabetic neuropathy.

20. Known or suspected history of alcohol or substance abuse.

21. Mental incapacity, unwillingness, or language barrier precluding adequate understanding of or cooperation with the study.

22. Women of childbearing potential who are pregnant, breast-feeding, or intend to become pregnant. Women of childbearing potential must have a negative pregnancy test at Screening and must agree to use adequate contraceptive methods during the study and for 1 additional menstrual cycle following the end-of treatment visit (see inclusion criterion 5).

23. History of allergy or sensitivity to M1 antagonists or any of the components of the investigational product formulations.

24. Known allergy or hypersensitivity to pirenzepine or another component of the investigational product.

25. History of sensitive skin, as defined by a requirement to use soap and skin products formulated for “sensitive skin”.

26. Currently taking any medicines to treat overactive bladder (anticholinergic agents, such as Gelnique).

27. Failure or inability to perform screening or baseline assessments.

28. Patients with any condition that could potentially interfere with the conduct of the study or confound efficacy evaluations, including the following as specified in numbers 29 through 35 below:

29. Pain or neuropathy from another cause (including central pain, radiculopathy, painful arthritis, etc).

30. Skin or soft-tissue lesions in the area affected by neuropathy that are painful or could alter sensation.

31. Systemic infections (eg, HIV, hepatitis, tuberculosis, syphilis).

32. Exposure to an experimental drug, experimental biologic, or experimental medical device within 3 months before screening.

33. Any open wound(s) and/or sunburn(s) in the dosing area. Subjects who have a wound and/or sunburn at screening that is anticipated to resolve before day -1 can be enrolled.

34. History of a serious skin disease (as determined by the Investigator), such as skin cancer, psoriasis, or eczema.

35. Receipt of a tattoo in the dosing area within 12 months of dosing.
